# A Novel Sperm-Delivered Toxin Causes Late-Stage Embryo Lethality and Transmission Ratio Distortion in *C. elegans*

**DOI:** 10.1371/journal.pbio.1001115

**Published:** 2011-07-26

**Authors:** Hannah S. Seidel, Michael Ailion, Jialing Li, Alexander van Oudenaarden, Matthew V. Rockman, Leonid Kruglyak

**Affiliations:** 1 Department of Ecology and Evolutionary Biology, Princeton University, Princeton, New Jersey, United States of America; 2 Lewis-Sigler Institute for Integrative Genomics, Princeton University, Princeton, New Jersey, United States of America; 3 Department of Biology, University of Utah, Salt Lake City, Utah, United States of America; 4 Department of Physics, Massachusetts Institute of Technology, Cambridge, Massachusetts, United States of America; 5 Department of Biology, Massachusetts Institute of Technology, Cambridge, Massachusetts, United States of America; 6 Department of Biology, New York University, New York, New York, United States of America; 7 Center for Genomics and Systems Biology, New York University, New York, New York, United States of America; 8 Howard Hughes Medical Institute, Chevy Chase, Maryland, United States of America; University of Bath, United Kingdom

## Abstract

The evolutionary fate of an allele ordinarily depends on its contribution to host fitness. Occasionally, however, genetic elements arise that are able to gain a transmission advantage while simultaneously imposing a fitness cost on their hosts. We previously discovered one such element in *C. elegans* that gains a transmission advantage through a combination of paternal-effect killing and zygotic self-rescue. Here we demonstrate that this element is composed of a sperm-delivered toxin, *peel-1*, and an embryo-expressed antidote, *zeel-1*. *peel-1* and *zeel-1* are located adjacent to one another in the genome and co-occur in an insertion/deletion polymorphism. *peel-1* encodes a novel four-pass transmembrane protein that is expressed in sperm and delivered to the embryo via specialized, sperm-specific vesicles. In the absence of *zeel-1*, sperm-delivered PEEL-1 causes lethal defects in muscle and epidermal tissue at the 2-fold stage of embryogenesis. *zeel-1* is expressed transiently in the embryo and encodes a novel six-pass transmembrane domain fused to a domain with sequence similarity to *zyg-11*, a substrate-recognition subunit of an E3 ubiquitin ligase. *zeel-1* appears to have arisen recently, during an expansion of the *zyg-11* family, and the transmembrane domain of *zeel-*1 is required and partially sufficient for antidote activity. Although PEEL-1 and ZEEL-1 normally function in embryos, these proteins can act at other stages as well. When expressed ectopically in adults, PEEL-1 kills a variety of cell types, and ectopic expression of ZEEL-1 rescues these effects. Our results demonstrate that the tight physical linkage between two novel transmembrane proteins has facilitated their co-evolution into an element capable of promoting its own transmission to the detriment of organisms carrying it.

## Abstract 2

A sperm-delivered toxin and an embryo-expressed antidote form a co-adapted gene complex in C. elegans that promotes its own transmission to the detriment of organisms carrying it.

## Author Summary

Natural selection typically favors only those genetic variants that increase the overall fitness of the organism. Occasionally, however, variants arise that are able to increase their representation in future generations, while simultaneously reducing the fertility or fecundity of their hosts. Although such variants occur in a wide variety of taxa, their genetic bases and molecular mechanisms remain poorly understood. Here we demonstrate that one such variant in the roundworm *C. elegans* is composed of two adjacent genes: a sperm-delivered toxin and an embryo-expressed antidote. The toxin protein is expressed in sperm and delivered to the embryo upon fertilization. In the presence of the toxin, embryos that don't inherit the antidote gene die during late embryogenesis, whereas those that do develop normally. Both the toxin and the antidote genes encode transmembrane proteins, and both are evolutionarily novel. Our results imply that the tight physical linkage between these two novel genes has facilitated their evolution into a co-adapted gene complex capable of promoting its own transmission to the detriment of host fitness.

## Introduction

The evolutionary fate of an allele ordinarily depends on the reproductive fitness of the organisms carrying it. In some cases, however, alleles are able to increase their representation in future generations while being neutral or detrimental to the fitness of their bearers. These elements, sometimes termed “selfish” or “parasitic” genes, influence transmission probabilities in a variety of ways. Some self-replicate and insert themselves into new genomic locations (e.g., transposons) [1]. Others act during meiosis to preferentially segregate into the oocyte [2]–[4] or to reduce the viability of sperm or spores inheriting alternate alleles [5]–[8]. Still others act at the level of the zygote to destroy progeny not inheriting them. *Medea*-factors in *Tribolium* destroy non-carrier animals through a combination of maternal-effect killing and zygotic self-rescue [9]. An analogous phenomenon occurs in organisms infected by the maternally transmitted bacteria, *Wolbachia* or *Cardinium* [10]–[12], which modify the sperm of infected males to cause lethal defects when paired with the oocytes of uninfected females.

Previously, we discovered a nuclear-encoded element in *Caenorhabditis elegans* that kills non-carrier animals in a novel way [13]. This element, referred to as the *peel-1/zeel-1* element, is polymorphic within the species, and when animals carrying the *peel-1/zeel-1* element are crossed to animals lacking it, the *peel-1/zeel-1* element acts in the F1 heterozygote via paternal effect to kill F2 or backcross embryos not inheriting it. This lethality acts independently of maternal genotype and causes the *peel-1/zeel-1* element to become over-represented among the viable progeny of heterozygous sires, even while incurring a substantial fitness cost to these animals.

Paternal-effect loci are extremely rare in all of biology [14],[15], and the observed combination of nuclear-encoded, paternal-effect killing and zygotic self-rescue is unprecedented. In *C. elegans*, moreover, a paternal-effect locus whose effects can be rescued zygotically is mechanistically surprising because in this species, sperm-supplied factors are thought to act only during fertilization and first cleavage [16], whereas zygotic transcription does not begin until the four-cell stage [17].

Although the *peel-1/zeel-1* element is capable of promoting its own transmission, it rarely has the opportunity to do so in natural populations. *C. elegans* is an androdioecious species that reproduces primarily through self-fertilizing hermaphrodites. Because inbreeding is high [18], the *peel-1/zeel-1* element normally exists in the homozygous state, where no opportunity for self-promotion exists.

High inbreeding notwithstanding, out-crossing events in *C. elegans* between hermaphrodites and males do occur, albeit rarely [18]–[20]. And because the *peel-1/zeel-1* element is globally distributed and confers no apparent fitness disadvantage in the homozygous state [13], this element is expected to drive itself to fixation faster than a neutrally evolving locus. Consistent with this prediction, in laboratory populations where out-crossing is forced, the *peel-1/zeel-1* element fixes rapidly [13]. In natural populations, however, the *peel-1/zeel-1* element has remained polymorphic for an estimated 8 million generations [13], much longer than expected under neutrality. One likely explanation for this paradox is that the *peel-1/zeel-1* element is tightly linked to a polymorphism maintained by balancing selection, and the tightness of this linkage maintains the *peel-1/zeel-1* element in the polymorphic state [13].

Given the unusual genetics of the *peel-1*/*zeel-1* element, we sought to understand its mechanism of action. We previously identified one component of the *peel-1/zee1-1* element as the gene *zeel-1* (*Y39G10AR.5*), which acts zygotically to rescue the paternal-effect killing [13]. Here we demonstrate that *zeel-1* is fully separable from the paternal-effect killing, and that this killing activity is encoded by a second gene, *peel-1* (*Y39G10AR.25*). We show that PEEL-1 acts as a sperm-supplied toxin, and ZEEL-1 an embryo-expressed antidote. We characterize the developmental defects caused by sperm-supplied PEEL-1, and we report a dose-dependent relationship between the severity of these defects and the quantity of PEEL-1 delivered to the embryo. We analyze the phylogenetic origins and functionality of each domain of *zeel-1*, and we test the tissue-autonomy of *zeel-1* rescue. Finally, in order to determine whether *peel-1* and *zeel-1* can function outside of embryogenesis, we express both genes ectopically in adults.

## Results

### *peel-1* and *zeel-1* Are Genetically Distinct

The genetics of the *peel-1/zeel-1* element are consistent with it being composed of two interacting loci: a dominant-lethal, paternal-effect “toxin,” *peel-1*, and a zygotically acting “antidote,” *zeel-1* [13]. The activities of *peel-1* and *zeel-1* are present in the reference strain, Bristol (N2), and in approximately two-thirds of wild isolates [13]. These strains are said to carry the *peel-1/zeel-1* element. The commonly used wild strain, collected from Hawaii (CB4856), and all but two of the additional wild strains lack the activities of both *peel-1* and *zeel-1* [13]. The two remaining strains, one collected from Germany (MY19) and one collected from Utah (EG4348), exhibit the activity of *zeel-1* but are unable to induce the paternal-effect killing ([13], Figure S1A).

We previously mapped the *peel-1/zeel-1* element in the Bristol strain to a 62 kb interval on the left arm of chromosome I [13]. Within this interval, we identified a single gene capable of providing antidote activity. This gene, which we named *zeel-1*, encodes a 917-amino acid protein whose *N*-terminus is predicted to form a six-pass transmembrane domain and whose *C*-terminus exhibits sequence similarity to ZYG-11, a substrate-recognition subunit of an E3 ubiquitin ligase [21]. The Hawaii strain carries a 19 kb deficiency (*niDf9*) spanning *zeel-1*, and this deficiency is shared by all other wild isolates lacking the activities of both *peel-1* and *zeel-1* [13]. This deficiency is not shared by wild strains carrying the *peel-1/zeel-1* element, nor by MY19 or EG4348 ([13], unpublished data).

**Figure 1:**
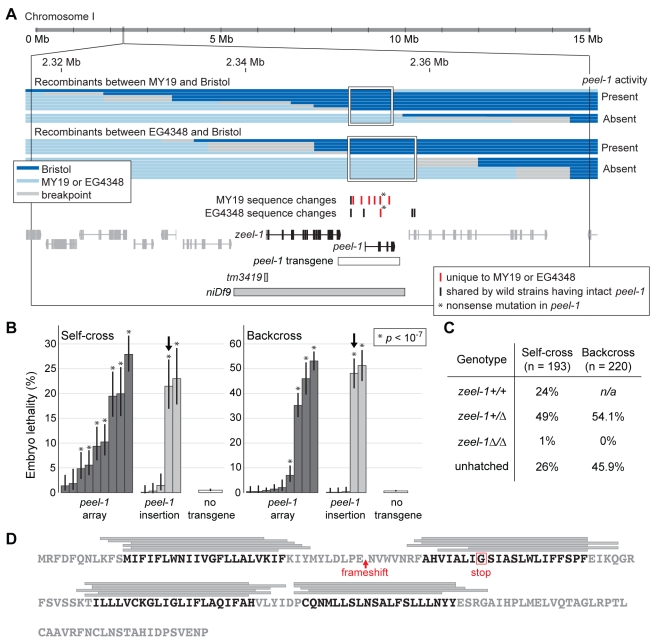
*peel-1* is located adjacent to *zeel-1* and encodes a four-pass transmembrane protein. (A) The large black box outlines the genomic region to which *peel-1* was mapped previously [13]. Horizontal bars represent the recombinants used to map the *peel-1* mutations in strains MY19 and EG4348. The maximum intervals defined by these mapping experiments are outlined by white boxes. Adjoining breakpoints are excluded because they contain no sequence changes relative to Bristol. Below the recombinants, tick marks indicate all sequence changes in MY19 or EG4348 located within the boxed intervals. Horizontal bars represent the *peel-1* transgene, the *zeel-1(tm3419)* deletion allele, and the deficiency, *niDf9*. *niDf9* is carried by the Hawaii strain and by all other wild isolates lacking the activities of both *peel-1* and *zeel-1* [13]. (B) The Bristol allele of the *peel-1* transgene shown in (A) was tested for its ability to restore *peel-1* activity to animals carrying the *peel-1* nonsense mutation found in EG4348. To test for *peel-1* activity, transgenic animals were crossed to the Hawaii strain, and embryo lethality was scored from self-fertilizing F1 hermaphrodites (self-cross) and F1 males backcrossed to Hawaii hermaphrodites (backcross). Nine independent extra-chromosomal arrays and five independent single-copy genomic insertions were tested. For each array or insertion, 200 to 650 embryos were scored per self-cross or backcross. Ten control replicates were performed in parallel, each including 200 to 400 embryos. The global mean of these replicates is shown by the “no transgene” bars, and lethality for each individual replicate was less than 1.5%. Error bars indicate 95% binomial confidence intervals, calculated using the Agresti-Coull method [77]. * *p*<10^−7^, one-tailed binomial test relative to the mean of the control replicates. The observed variability among extra-chromosomal arrays was expected because of germline silencing [30]. The three single-copy insertions showing no *peel-1* activity probably represent incomplete insertion events, which are a common outcome of the MosSCI method [59]. Arrows indicate the insertion used for further analysis in (C). (C) To confirm that the lethality observed in (B) was limited to *zeel-1(Δ)* embryos, an additional self-cross and backcross were performed using the insertion marked in (B). All hatched progeny were genotyped at *zeel-1*. In both crosses, the genotype frequencies among surviving progeny differed significantly from their Mendelian expectations (χ^2^ tests, *p*<10^−9^). *n/a*, not applicable. (D) The amino acid sequence of PEEL-1. Grey bars indicate predicted transmembrane helices, as predicted by (from top to bottom): TopPred [71], Tmpred [72], TMHMM [73], SOSUI [74], PHDhtm [75], and HMMTOP [76]. Regions predicted by at least four algorithms are highlighted in black. The frameshift in MY19 and the stop codon in EG4348 are indicated in red.

The phenotype of MY19 and EG4348 demonstrates that the *zeel-1* gene is not sufficient for *peel-1* activity. Conversely, a deletion allele of *zeel-1* in the Bristol background demonstrates that *zeel-1* is also not required for it. This deletion, *tm3419*, removes 221 base pairs spanning the start codon of *zeel-1* (Figure 1A). As expected, this deletion abolishes antidote activity (Figure S1B); however, this deletion does not abolish the paternal-effect killing (1B). *zeel-1* is therefore genetically separable from a second, paternal-effect locus, *peel-1*. As a consequence of this separability, *zeel-1* deletions in the Bristol and Hawaii backgrounds have opposite phenotypic effects: The *niDf9* deficiency is perfectly viable, whereas the *tm3419* deletion behaves as a conventional recessive-lethal mutation.

### *peel-1* Encodes a Novel, Four-Pass Transmembrane Protein of Unknown Function

In MY19 and EG4348, absence of *peel-1* activity is tightly linked to the 62 kb *peel-1* interval ([13], Figure S1C), suggesting that these strains carry loss-of-function alleles of *peel-1*, rather than extra-genic suppressors. In addition, sequence analysis of the *peel-1* interval in MY19 [13] and EG4348 (see Materials and Methods) indicates that absence of *peel-1* activity in these strains is not caused by recombination breaking apart the *peel-1/zeel-1* element. We hypothesized, therefore, that MY19 and EG4348 carry secondary, loss-of-function mutations in *peel-1* We reasoned that by identifying these mutations, we would be able to identify *peel-1* itself.

To accomplish this goal, we crossed MY19 and EG4348 to a strain of the Bristol background and generated recombinant chromosomes across the *peel-1* interval. Using these recombinants, we mapped the causative mutations in MY19 and EG4348 to regions of less than 10 kb (Figure 1A). We then sequenced these regions to identify all sequence changes relative to Bristol. After excluding those sequence changes shared by one or more wild strains having intact *peel-1* activity (Figure 1A, Figure S2), we defined six candidate mutations in MY19 and a single candidate mutation in EG4348.

The candidate mutations in MY19 and EG4348 reside in the intergenic interval immediately downstream of *zeel-1* (Figure 1A). We searched for genes in this interval using targeted RT-PCR on Bristol animals, and we discovered a previously unannotated transcript. This transcript encodes a 174-amino acid protein (Figure 1A,D), and three observations confirm this gene to be *peel-1*. First, the single candidate mutation in EG4348 and one of the candidate mutations in MY19 produce nonsense mutations in this transcript, consistent with the phenotype of these strains (frameshift in MY19; glycine to stop codon in EG4348) (Figure 1A,D; Figure S2). Second, this gene resides within the 19 kb deficiency carried by the Hawaii strain and in all other wild isolates lacking the activities of both *peel-1* and *zeel-1* (Figure 1A). Third, when we expressed the Bristol allele of this gene transgenically in a strain carrying the nonsense mutation found in EG4348, *peel-1* activity was restored (Figure 1B–C).

PEEL-1 is a hydrophobic protein containing four predicted transmembrane helices (Figure 1D). Neither the peptide nor the nucleotide sequence of *peel-1* has any detectable sequence similarity to any other gene in *C. elegans* or in the GenBank sequence database. Although *peel-1* and *zeel-1* are located adjacent to one another in the genome and oriented in the same direction, we were unable to recover transcripts carrying both genes (unpublished data), demonstrating that *peel-1* and *zeel-1* are not isoforms of a single transcript or cistrons in an operon. We conclude that the *peel-1/zeel-1* element is composed of a 19 kb insertion carrying two distinct genes: *peel-1*, which kills embryos via paternal-effect, and *zeel-1*, which acts zygotically to rescue this lethality.

Henceforth we refer to the Bristol alleles of *peel-1* and *zeel-1* as *peel-1(+)* and *zeel-1(+)* and the Hawaii alleles as *peel-1(*Δ*)* and *zeel-1(*Δ*)*. We use the term “*peel-1*-affected embryos” to refer to *zeel-1(*Δ*)* embryos fathered by a *peel-1(+)* animal.

### *peel-1* Is Expressed in Spermatocytes, and the Protein Localizes to Fibrous Body-Membranous Organelles

**Figure 2:**
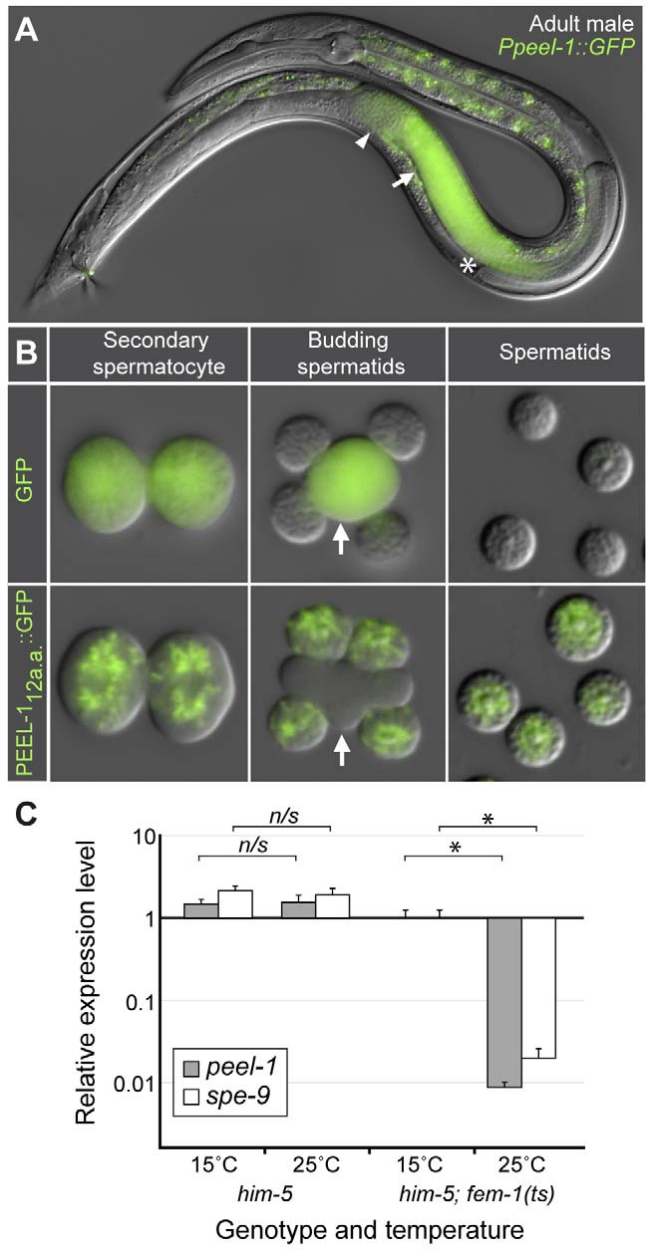
*peel-1* is expressed exclusively in sperm and carries an *N*-terminal sperm localization signal. (A) Adult male expressing *Ppeel-1::GFP*. The tube-like gonad begins at the asterisk, extends toward the head, folds over on itself, and then extends toward the tail. The arrow and arrowhead indicate spermatocytes and sperm, respectively. Fluorescence outside the gonad is auto-fluorescence. Nomarski and fluorescence channels are overlaid. (B) Secondary spermatocytes, budding spermatids, and mature spermatids expressing either untagged GFP or GFP tagged with the *N*-terminal 12 amino acids of PEEL-1 *(PEEL-1_12a.a._::GFP)*. Arrows indicate residual bodies. Nomarski and fluorescence channels are overlaid. (C) Relative expression levels of *peel-1* and *spe-9* in *him-5(e1490)* and *him-5(e1490) fem-1(hc17ts)* adult males at the permissive (15°C) and restrictive (25°C) temperatures. *him-5(e1490)* was included to aid in collection of males. Expression levels were calculated relative to the *him-5(e1490) fem-1(hc17ts)* 15°C sample. Runs were performed in triplicate and standard deviations are shown. * *p*<10^−5^, one-tailed Student's *t* test on the normalized expression levels. *n/s*, *p*>0.05.

As expected for a paternal-effect gene, *peel-1* is expressed exclusively in sperm. In both males and hermaphrodites, a GFP reporter driven by the *peel-1* promoter was expressed strongly in spermatocytes but not in any other tissue (Figure 2A). In *fem-1(ts)* mutants, which lack sperm [22], *peel-1* expression via quantitative RT-PCR was undetectable in hermaphrodites and over 100-fold reduced in males (Figure 2C). Residual expression in males probably reflects incomplete penetrance of the *fem-1(ts)* allele, because *fem-1(ts)* males occasionally produce a small number of sperm [22], and another sperm-specific gene, *spe-9* [23], also showed residual expression in males (Figure 2C).

The sperm-specific expression of *peel-1* suggested that the paternal-effect killing occurs through delivery to the embryo of either sperm-supplied *peel-1* transcripts or sperm-supplied PEEL-1 protein. To distinguish between the two, we searched for *peel-1* transcripts in mature sperm via single-molecule fluorescence in situ hybridization (FISH). *peel-1* transcripts were observed in spermatocytes but not in mature sperm (Figure S3), thus excluding such transcripts as the likely mediators of *peel-1* toxicity.

**Figure 3:**
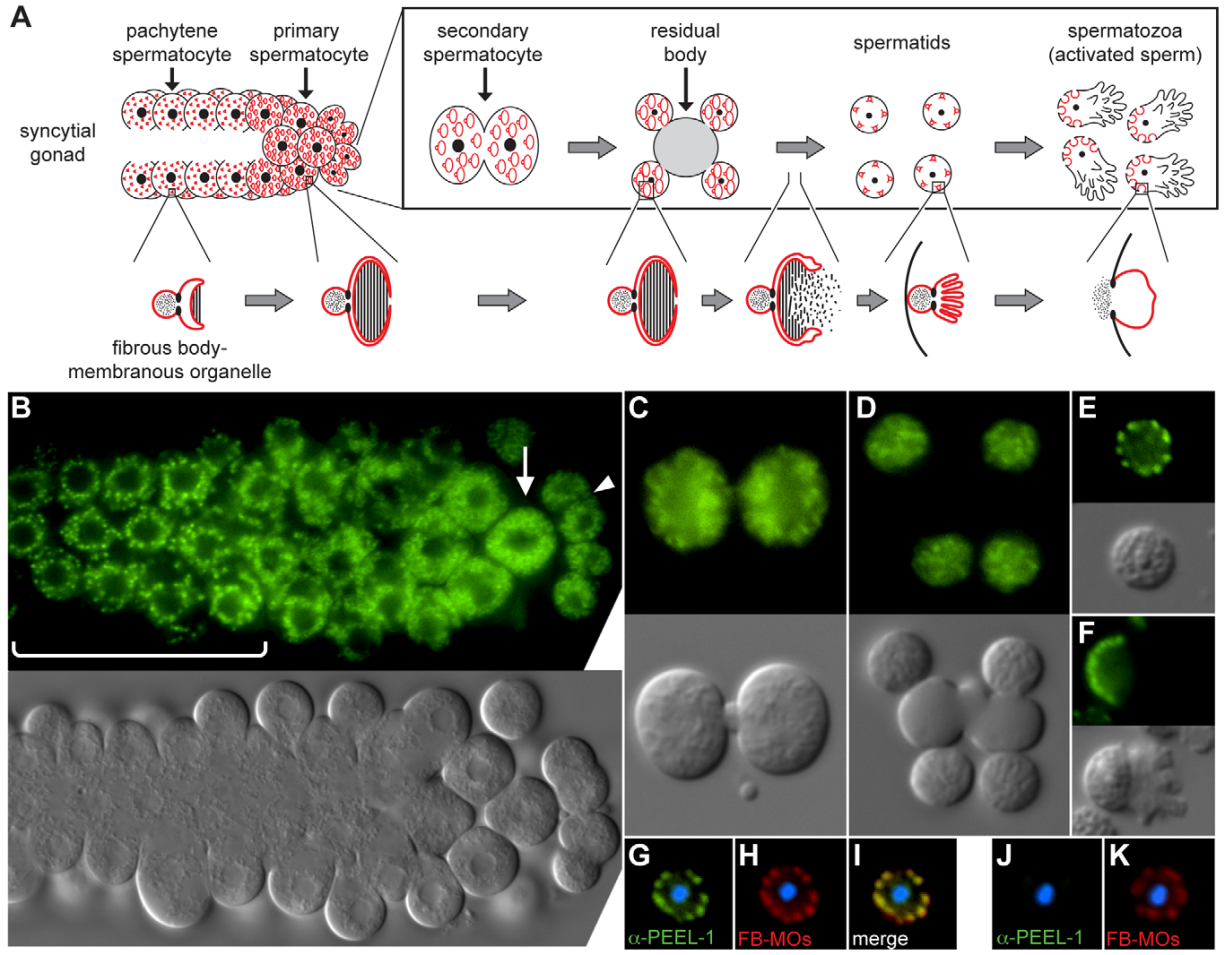
PEEL-1 localizes to fibrous body-membranous organelles. (A) Diagram of spermatogenesis and fibrous body-membranous organelle (FB-MO) development, adapted with permission from [16]. FB-MOs develop in pachytene spermatocytes as membrane-bound organelles having a head region separated by a collar-like constriction from a set of membrane folds (lower panel; red). As spermatogenesis proceeds, the membrane folds grow and extend into arm-like protrusions, enveloping bundles of polymerized Major Sperm Protein, referred to as fibrous bodies (hatched region). Coincident with budding of spermatids from the residual body, the membrane folds of FB-MOs retract, and the fibrous bodies depolymerize into the cytoplasm. The FB-free MOs then move to a position just inside the plasma membrane, and upon sperm activation, they fuse with the plasma membrane opposite the pseudopod. (B–F) Nomarski and fluorescence images of spermatocytes and sperm expressing *PEEL-1::GFP*. Panels in (B) show the proximal arm of a male gonad, oriented with pachytene spermatocytes towards the left (bracketed region). Arrow and arrowhead indicate primary and secondary spermatocytes, respectively. Panels in (C–F) show higher resolution images of the following stages: secondary spermatocyte (C), budding spermatids (D), unactivated spermatid (E), and activated spermatozoan (F). (G–K) Spermatids were dissected from *peel-1(+)* (G–I) or *peel-1(Δ)* (J–K) males and stained with anti-PEEL-1 (green) and the FB-MO marker, 1CB4 (red) [64]. Nuclei are stained with DAPI (blue).

Next, we searched for PEEL-1 protein both by tagging PEEL-1 with GFP and by staining sperm with an antibody raised against the *C*-terminal 15 amino acids of PEEL-1. Both experiments demonstrate that PEEL-1 protein is packaged into sperm, and this packaging is mediated by localization of PEEL-1 to sperm-specific vesicles called fibrous body-membranous organelles (FB-MOs) (Figure 3A). PEEL-1::GFP was visible in mature sperm (Figure 3E–F), and at each stage of spermatogenesis, its localization matched the pattern expected for a protein located in the membranes of FB-MOs: In early spermatocytes, PEEL-1::GFP localized to cytoplasmic puncta (Figure 3B), but after the pachytene stage, these puncta dissociated into a mesh-like web (Figure 3B–D); after the completion of spermatogenesis, PEEL-1::GFP re-condensed into puncta located at the spermatid cortex (Figure 3E); and after sperm activation, these puncta localized opposite the newly formed pseudopod (Figure 3F). This localization pattern was replicated by the anti-PEEL-1 antibody (Figure 3G), and staining with this antibody overlapped perfectly with a marker of FB-MOs (Figure 3H–I).

We also discovered that the leader peptide of PEEL-1 can act as a sperm-localization signal. Our *Ppeel-1::GFP* reporter, which expressed untagged GFP, showed diffuse localization in spermatocytes and was excluded from sperm (Figure 2B). This exclusion was not surprising because GFP is a heterologous protein, and trafficking of cellular components into sperm is tightly regulated [16]. Nevertheless, when we tagged GFP with the *N*-terminal 12 amino acids of PEEL-1 (MRFDFQNLKFSM), its localization changed dramatically. The tagged version of GFP localized to a reticulated structure within spermatocytes, and this structure was trafficked into sperm (Figure 2B). To our knowledge, this result represents the first identification of a sperm localization signal in *C. elegans*.

### *peel-1*-Affected Embryos Show Late-Stage Defects in Muscle and Epidermal Tissue

**Figure 4:**
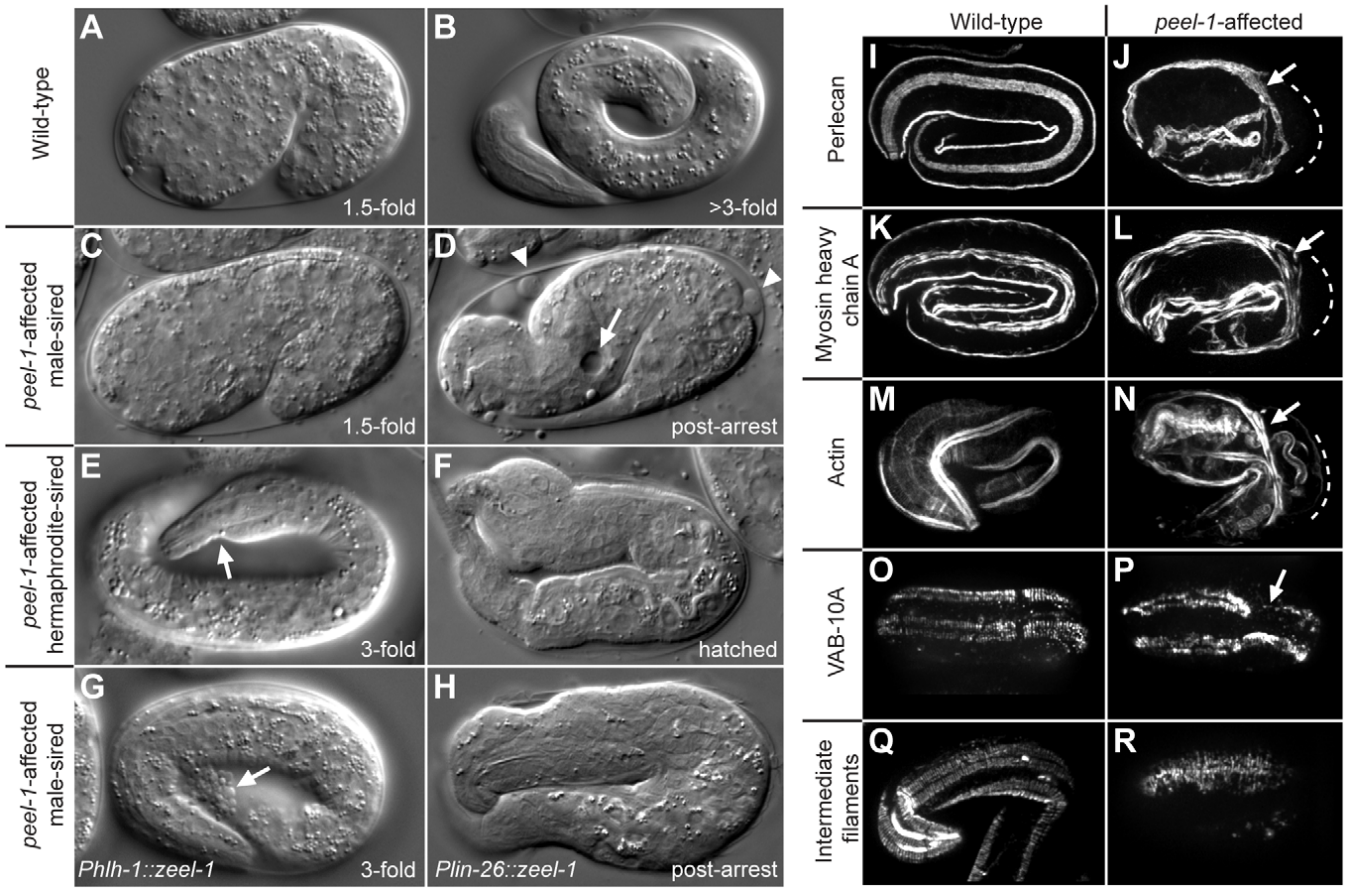
*peel-1*-affected embryos exhibit late-occurring defects in muscle and epidermal tissue. (A and B) Wild-type embryo at the 1.5-fold stage (A) and just before hatching (B). (C and D) *peel-1*-affected, male-sired embryo at the 1.5-fold stage (C) and approximately 4 h after the 2-fold arrest (D). Relative to its shape at the 2-fold stage, the embryo in (D) has shortened longitudinally and thickened circumferentially. Thinning of the tail, distention of the excretory cell (arrow), and epidermal leakage (arrowheads) are visible. (E and F) *peel-1*-affected, hermaphrodite-sired embryos displaying less severe phenotypes than the embryo shown in (D). In (E), the embryo has elongated past the 2-fold stage, but muscle detachment is visible (arrow). In (F), the embryo has hatched but is severely deformed. (G–H) *peel-1*-affected, male-sired embryos expressing *zeel-1* in only muscle (G) or only in epidermis (H). The embryo in (G) has elongated past the 2-fold stage, but epidermal leakage is visible (arrow). The embryo in (H) has arrested paralyzed at the 2-fold stage but has survived to hatching. (I–N) Perlecan, myosin heavy chain A, and F-actin were visualized in wild-type and *peel-1*-affected, male-sired embryos. In *peel-1*-affected embryos, muscle detachment is evident at the mid-embryo bend, where muscle fibers (arrows) are displaced inward relative to their proper locations (dashed lines). (O–R) VAB-10A and intermediate filaments were visualized in wild-type and *peel-1*-affected, male-sired embryos. In *peel-1*-affected embryos, VAB-10A and intermediate filaments are recruited properly to the four muscle quadrants, but they do not organize into evenly spaced, circumferentially oriented bands. In (P), VAB-10A staining is absent in one dorsal quadrant at the mid-embryo bend (arrow). Images in (O–P) are dorsal views.

Unlike other known paternal-effect genes [14],[15], sperm-supplied PEEL-1 does not cause defects until late in development. In *peel-1*-affected embryos, early embryogenesis, gastrulation, epidermal enclosure, and early elongation occur normally (Figure 4C). Then, at the 2-fold stage of elongation, when all major tissues have begun differentiating and nearly all embryonic cell divisions have already occurred, the majority of *peel-1*-affected embryos arrest elongation and fail to begin rolling within their eggshells (Movies S1–S3). Shortly thereafter, the bulk of the embryo compresses inward, towards the mid-embryo bend, and the head and tail become flaccid and thin (Figure 4D, Movies S1–S2). Approximately 2 h later, cytoplasm begins leaking from the external epidermis, and the lumen of the excretory cell distends to form large vacuoles (Figure 4D, Movies S1–S2).

The defects observed in *peel-1*-affected embryos indicate severe malfunction of muscle and epidermal tissue. The phenotype of paralysis and 2-fold arrest is characteristic of a complete absence of the function of body-wall muscle [24],[25]. The shape changes observed after the 2-fold arrest indicate detachment of body-wall muscle fibers from the overlying epidermis [26]. Epidermal leakage and distention of the excretory cell, the only epidermal cell located in the interior of the animal, indicate further deterioration of both external and internal epidermis. These four defects—paralysis and 2-fold arrest, muscle-epidermal detachment, epidermal leakage, and excretory cell distention—are not known to occur as side-effects of one another [25],[27], suggesting that sperm-supplied PEEL-1 may affect each tissue independently.

Paralysis and 2-fold arrest have only two known causes: defective sarcomere assembly and lack of muscle contraction [24],[25]. To distinguish between the two, we examined (*i*) the localization of perlecan, a basement membrane protein required for sarcomere recruitment [25], and (*ii*) the structure of actin and myosin myofilaments, which assemble downstream of all other sarcomere proteins [24]. In *peel-1*-affected embryos, perlecan localized normally (Figure 4I–J). Likewise, actin and myosin filaments assembled correctly, except for slight abnormalities in regions of muscle detachment (Figure 4K–N). We conclude that in *peel-1*-affected embryos, the phenotype of paralysis and 2-fold arrest results from a defect in muscle contraction, not sarcomere assembly.

Next, to determine the cause of muscle-epidermal detachment, we examined trans-epidermal attachments, the specialized structures that span the epidermal syncytium and anchor it to underlying muscle [27]. A weakening of these structures is known to cause muscle-epidermal detachment [28], and consistent with this causality, in *peel-1*-affected embryos these structures were highly disorganized. As visualized by VAB-10A and intermediate filaments, trans-epidermal attachments did not organize into evenly spaced, circumferentially oriented bands. Instead, these bands were clumpy, disordered, and non-uniform in width (Figure 4O–R). This disorganization occurred even in areas where muscles remained attached, suggesting it to be the cause of muscle detachment, rather than an effect of it. In addition, in areas of highest stress, such as the mid-embryo bend, staining in post-arrest embryos (but not pre-arrest embryos) was often absent altogether (Figure 4P). This absence implies that trans-epidermal attachments in *peel-1*-affected embryos are so weak that in areas of highest stress, they rupture entirely.

### *peel-1* Toxicity Is Dose-Dependent

**Figure 5:**
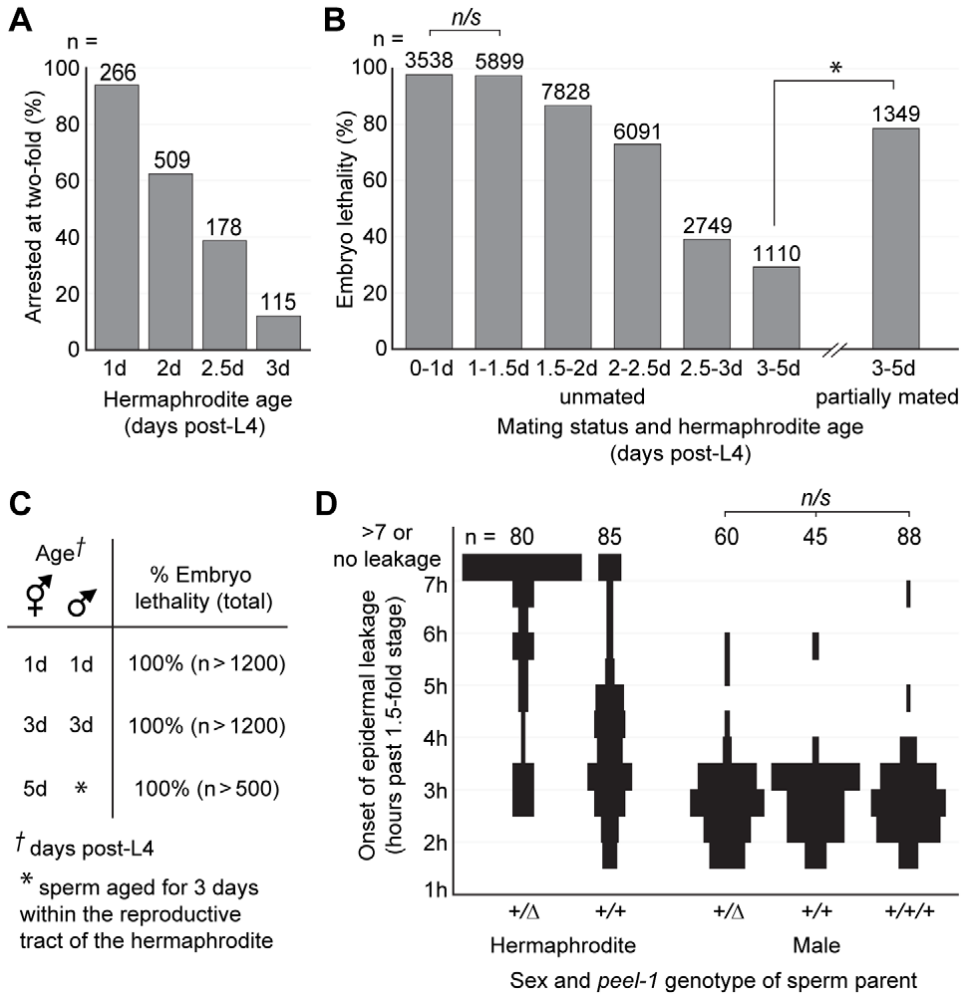
The phenotypic effects of sperm-supplied PEEL-1 are dose-dependent. (A) The proportion of embryos arresting at the 2-fold stage was calculated among *peel-1*-affected embryos sired by 1- to 3-d-old hermaphrodites. Within each age class, embryos derive from a total of approximately 50 to 150 hermaphrodites. All pair-wise combinations of age classes were compared using χ^2^ tests. For all pairs, *p*<10^−5^. (B) Embryo lethality was scored among *peel-1*-affected embryos sired by unmated, 1- to 5-d-old hermaphrodites and by partially mated, 3- to 5-d-old hermaphrodites. In the unmated experiment, 91 hermaphrodites were followed from the onset of adulthood, and all embryos laid during the first 5 d of adulthood were scored. The results for 10 randomly selected broods are shown in Figure S4. To generate embryos sired by partially mated animals, 130 hermaphrodites were briefly mated to males following the L4 molt, and embryos were collected during days 3 to 5 from the 35 hermaphrodites that produced a mixture of self- and cross-progeny. Self- and cross-progeny were distinguished by the use of an integrated GFP marker carried by the male, and cross-progeny were excluded from analysis. χ^2^ tests were used to compare the unmated and partially mated *“3–5d”* age classes, as well as all pair-wise combinations of age classes within the unmated experiment. *n/s*, *p*>0.05. * and all unlabeled pairs within the unmated experiment, *p*<10^−5^. (C) Embryo lethality was scored among *peel-1*-affected embryos derived from crosses between (i) 1-d-old males and hermaphrodites, (ii) 3-d-old males and hermaphrodites, and (iii) 5-d-old hermaphrodites that had been removed from males on day 2 in order to allow male sperm to age for 3 d within the reproductive tract of the hermaphrodite. In each cross, embryos derive from a total of 12 to 16 hermaphrodites. (D) Spindle plots showing the onset of epidermal leakage in *peel-1*-affected embryos sired by hermaphrodites carrying one or two copies of *peel-1(+)*, or by males carrying one, two, or three copies of *peel-1(+)*. A third copy of *peel-1* was added using the single-copy insertion of the *peel-1* transgene marked in Figure 1B. The width of each bar reflects the proportion of embryos initiating leakage in each time interval. All pair-wise combinations of spindle plots were compared using Mann-Whitney U tests on the raw distributions of leakage times. *n/s*, *p*>0.05. For all other pairs, *p*<10^−5^.

Although the majority of *peel-1*-affected embryos display the aforementioned defects in muscle and epidermal tissue, the severity of these defects is variable and depends on the sex [13] and age of the sperm parent. Male-sired embryos always arrest paralyzed at the 2-fold stage, always exhibit epidermal leakage, and never hatch (*n*>2,000). Some hermaphrodite-sired embryos, on the other hand, elongate past the 2-fold stage (Figure 4E) or do not exhibit epidermal leakage. Occasionally, hermaphrodite-sired embryos even hatch, and the hatched progeny range from severely deformed larvae that die soon after hatching (Figure 4F) to morphologically normal larvae that develop into viable, fertile adults. In addition, among hermaphrodite-sired embryos, the proportion of *peel-1*-affected embryos arresting at the 2-fold stage and the proportion failing to hatch decreased dramatically with parental age (Figure 5A–B, Figure S4). Among male-sired embryos, parental age had no effect (Figure 5C).

One explanation for the decreased phenotypic severity of hermaphrodite- versus male-sired embryos is PEEL-1 dosage. Male sperm are up to 5-fold larger than hermaphrodite sperm [29], and as such, they may deliver more PEEL-1 protein to the embryo. In support of this hypothesis, we were able to alter the phenotype of *peel-1*-affected embryos, independent of sperm origin, by varying *peel-1* dosage. Among hermaphrodite-sired embryos, doubling *peel-1* copy number resulted in earlier onset of epidermal leakage (Figure 5D). Among male-sired embryos, expression of *peel-1* exclusively from extra-chromosomal arrays, which are subject to germline silencing [30], had the opposite effect. For three of the *peel-1* arrays shown in Figure 1B, 3%–10% of male-sired embryos elongated past the 2-fold stage and hatched into deformed larvae (*n*>150 embryos per array). When the same *peel-1* transgene was expressed from a single-copy genomic insertion, which is not silenced, no hatching was observed (Figure 1C).

Given the relationship between PEEL-1 dosage, sperm size, and phenotypic severity, we suspected that the age-related decrease in phenotypic severity among hermaphrodite-sired embryos might reflect underlying size differences and size-based competition among hermaphrodite sperm. Hermaphrodite sperm vary approximately 2-fold in size ([29], personal observations) and are produced in a single bout of spermatogenesis at the onset of adulthood. Larger sperm in *C. elegans* experience a competitive advantage because they are able to crawl faster to reach the oocyte [29],[31]. One explanation for the age effect, therefore, is that larger-than-average sperm monopolize fertilization events early in life, leaving smaller, less toxic sperm to dominate fertilizations later on. In support of this hypothesis, we were able to reduce the age effect among hermaphrodite-sired embryos by delaying the hermaphrodite's use of self-sperm via partial mating to a male (Figure 5B). This result demonstrates that the age effect arises from a correlation between the competitive ability of each sperm and its toxicity to the embryo. Given the known biology of *C. elegans* sperm, the most parsimonious explanation for this correlation is that larger hermaphrodite sperm both are more competitive and carry more PEEL-1 protein. This correlation might also arise from PEEL-1 having a direct effect on the competitive ability of each sperm, although we have no evidence for such an effect.

To our knowledge, the above results represent the first evidence of competition among hermaphrodite sperm in vivo, as well as the first evidence of naturally occurring differences in sperm size affecting embryonic development. Insofar as PEEL-1 levels scale with sperm size, the wide phenotypic variability among hermaphrodite-sired embryos implies that for low levels of PEEL-1, phenotypic severity is acutely sensitive to PEEL-1 dosage. By the same logic, however, the phenotypic homogeneity among male-sired embryos implies that above a certain threshold level of PEEL-1, phenotypic severity ceases to increase. In support of this threshold effect, doubling or tripling *peel-1* copy number among male-sired embryos did not produce a more severe phenotype, at least as measured by the onset of epidermal leakage (Figure 5D).

### *zeel-1* Is Expressed Transiently in the Embryo, and Tissue-Specific Expression of *zeel-1* Produces Tissue-Specific Rescue

**Figure 6:**
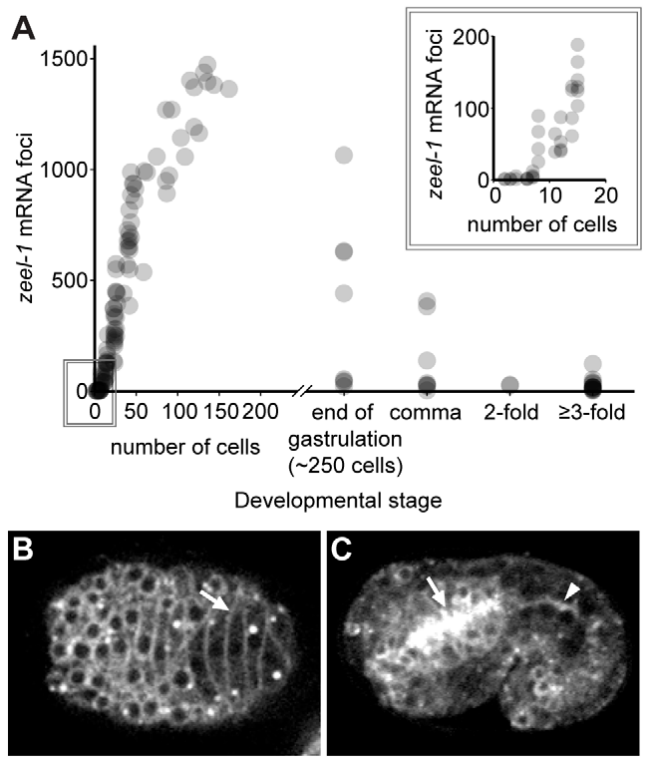
*zeel-1* is transiently expressed during embryogenesis and localizes to cell membranes. (A) *zeel-1* mRNAs were quantified in wild-type embryos via single-molecule fluorescence in situ hybridization [56]. Embryos were staged by counting nuclei manually (1- to 40-cell embryos), counting nuclei using image analysis software (41- to 200-cell embryos), or classifying embryos as end of gastrulation (∼250 cells), comma, 2-fold, or ≥3-fold. Inset shows a magnification of the boxed area. Each circle represents an independent embryo. *n* = 130. (B–C) Embryos expressing *ZEEL-1::GFP*. Panel in (B) shows a dorsal view during intercalation of epidermal cells. Arrow indicates an epidermal cell membrane. Panel in (C) shows a lateral cross-section of a 1.5-fold embryo. The apical face of the pharynx (arrow) and the intestine (arrowhead) are indicated.

Consistent with its function as an antidote to sperm-supplied PEEL-1, *zeel-1* is expressed in the embryo. Yet its expression is transient. By single-molecule FISH, *zeel-1* expression begins at the eight-cell stage, peaks at approximately the 150-cell stage, and then turns off (Figure 6A). Transient expression was also observed for a GFP-tagged version of ZEEL-1, whose levels peaked during mid-embryogenesis (Figure S5), but whose expression was not observed in late-stage embryos, nor larvae or adults (unpublished data).

Within embryos, ZEEL-1::GFP was expressed in all or almost all cell types (Figure 6B–C, Figure S5). The protein localized most strongly to cell membranes (Figure 6B–C), consistent with ZEEL-1 having an *N*-terminal transmembrane domain. In some tissues, such as the developing pharynx and intestine, ZEEL-1::GFP appeared more concentrated at the apical face (Figure 6C).

Ubiquitous *zeel-1* expression is consistent with *zeel-1*'s ability to rescue the seemingly independent muscle and epidermal defects observed in *peel-1*-affected embryos. To test the tissue-autonomy of *zeel-1* rescue, we expressed *zeel-1* only in muscle and only in epidermis. We used the promoters of *hlh-1* and *lin-26*, respectively, [32],[33], which initiate expression at the 80- to 100-cell stage [34]. Consistent with sperm-supplied PEEL-1 affecting muscle and epidermis independently, tissue-specific expression of *zeel-1* produced tissue-specific rescue. In male-sired embryos, expression of *zeel-1* only in muscle rescued the muscle defect of paralysis and 2-fold arrest, but it did not rescue epidermal leakage (*n* = 120 embryos; Figure 4G). (Muscle detachment and excretory cell distention could not be assayed because muscle movement, combined with epidermal leakage, ripped embryos apart entirely.) Conversely, expression of *zeel-1* only in the epidermis rescued the epidermal defects, but it did not rescue paralysis and 2-fold arrest (*n* = 69 embryos; Figure 4H). In hermaphrodite-sired embryos, both constructs fully rescued a subset of embryos, and rescue activity increased with hermaphrodite age (Figure S6). This result is consistent with the age effect among hermaphrodite-sired embryos and the fact that hermaphrodite-sired embryos do not always exhibit defects in both muscle and epidermal tissue.

### The Transmembrane Domain of *zeel-1* Is Evolutionarily Novel and Partially Sufficient for Antidote Activity

The structure of *zeel-1*—a *C*-terminal region (∼700 amino acids) predicted to be soluble and homologous to the highly conserved gene, *zyg-11*, and an *N*-terminus (∼200 amino acids) predicted to form a six-pass transmembrane domain—is highly unusual, and phylogenetic analysis indicates that this structure arose during a recent expansion of the *zyg-11* family. Most metazoan genomes contain one to two *zyg-11* orthologs, but in *C. elegans*, *C. briggsae*, and *C. remanei*, the *zyg-11* family has expanded such that these species carry 19 to 37 *zyg-11*-like genes each (Figure S7). Most of these additional family members probably arose after the split with out-group *C. japonica*, because the genome of *C. japonica* contains only three *zyg-11*-like genes (including *Cja-zyg-11* itself).

**Figure 7:**
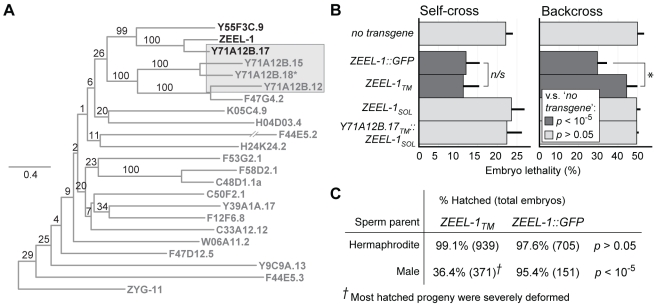
The transmembrane domain of *zeel-1* is evolutionarily novel and partially sufficient for function. (A) Maximum likelihood phylogeny of the protein sequences of all *zyg-11* homologs in *C. elegans*. Genes containing predicted transmembrane domains are highlighted in black. The transmembrane domains of these genes were excluded prior to analysis. Genes located in the tandem array are highlighted by a shaded grey rectangle. Scale bar indicates amino acid substitutions per site. Values on branches indicate percent bootstrap support. The asterisk indicates that the reference sequence of *Y71A12B.18* contains a single frame-shift, corrected prior to analysis. (B) Four *zeel-1*-derived transgenes were introduced into a strain carrying the *zeel-1* deficiency, *niDf9*, and tested for their ability to rescue *peel-1*-affected embryos. To test for rescue, transgenic animals were crossed to the Bristol strain, and lethality was scored among embryos derived from self-fertilizing F1 hermaphrodites (self-cross) and F1 males backcrossed to hermaphrodites of the original transgenic line (backcross). For each type of transgene, 4 to 13 independent extra-chromosomal arrays were tested. For each array, 100 to 600 embryos were scored per self-cross or backcross. Ten control replicates were performed in parallel, each including 100 to 400 embryos (“no transgene” bars). Among arrays or control replicates, lethality scores were averaged to obtain global means and standard deviations. Each transgene was tested for a reduction in lethality compared to the control replicates (Student's *t* tests, *p* values indicated by shading). Additionally, the two rescuing transgenes (*ZEEL-1_TM_* and *ZEEL-1::GFP*) were tested for significant differences relative to one another (Student's *t* tests; * *p*<0.005; *n/s*, *p*>0.05). For the rescuing transgenes, lethality was not reduced to zero because extra-chromosomal arrays are not transmitted to all progeny. (C) Hatch rates were compared between *peel-1*-affected embryos that we confirmed to have inherited either *ZEEL-1_TM_* or *ZEEL-1::GFP* (χ^2^ tests, *p* values shown). Separate comparisons were performed for male- and hermaphrodite-sired embryos. Unless otherwise specified, all hatched progeny appeared morphologically normal. Inheritance of the transgenes was determined by expression of the co-injection marker, *Pmyo-2::RFP*. Sibling embryos not inheriting the transgene were used as internal negative controls. The hatch rates of these controls were 2% (*n* = 601−653) among hermaphrodite-sired embryos and 0% (*n* = 341−933) among male-sired embryos.

Aside from *zeel-1*, only two other members of the *zyg-11* family—paralogs *Y71A12B.17* and *Y55F3C.9*—contain predicted transmembrane domains (Figures 7A, S6). The transmembrane domains of these three genes are homologous to one another, but these domains show no detectable sequence similarity to any other gene in *C. elegans* or in the GenBank sequence database. This pattern, combined with the closest-paralog relationship between the *C*-terminal domains of *zeel-1*, *Y71A12B.17*, and *Y55F3C.9* (Figures 7A, S6), implies that their shared transmembrane domain is evolutionarily novel and originated after the split between *C. elegans* and the *C. briggsae*/*C. remanei* lineage.

Analysis of gene order and sequence data suggests that *zeel-1* arose through duplication of the *Y71A12B.17* locus. *zeel-1* and *Y71A12B.17* are one another's closest paralogs (Figures 7A, S6), and the two genes are 55% identical at the amino acid level. *Y71A12B.17* is located 12 Mb from *zeel-1* in a tandem array of three additional *zyg-11* family members, none of which contain the *N*-terminal transmembrane domain. Assuming that *Y71A12B.17* and its neighbors originated in their current genomic location via repeated tandem duplication, then two scenarios for the origin of *zeel-1* and *Y71A12B.17* are possible. First, *Y71A12B.17* may have originated via duplication of another gene in the tandem array, gained its transmembrane domain during or after duplication, and later been duplicated again to produce *zeel-1*. Alternatively, the tandem array may have arisen through partial duplication of *Y71A12B.17*. The second scenario is less parsimonious than the first, because it requires secondary loss of the transmembrane domain during creation of the tandem array. However, the second scenario still implies that the *Y71A12B.17* locus predates *zeel-1*, because if the opposite were true, then *zeel-1* would form an out-group to the tandem array, and it does not (Figure 7A).

Given the chimeric structure of ZEEL-1, we tested whether either domain alone could rescue the lethality of *peel-1*-affected embryos. The *C*-terminal ZYG-11-like domain, ZEEL-1_SOL_, provided no rescue (Figure 7B). The transmembrane domain, ZEEL-1_TM_, provided full rescue to hermaphrodite-sired embryos, but only partial rescue to male-sired embryos (Figure 7B–C). In contrast, the positive control transgene, full-length ZEEL-1 tagged with GFP, provided full rescue to both male- and hermaphrodite-sired embryos (Figure 7B–C). We conclude that the transmembrane domain of ZEEL-1 is required for antidote activity, and that this domain alone is able to neutralize the low doses of PEEL-1 delivered by hermaphrodite sperm but not the higher doses delivered by male sperm.

The partial rescue activity of ZEEL-1_TM_ demonstrates that ZEEL-1_SOL_ does contribute to the antidote activity of full-length ZEEL-1. To examine this contribution more carefully, we tested whether ZEEL-1_SOL_ could rescue the lethality of *peel-1*-affected embryos when fused to the transmembrane domain of *zeel-1*'s closest relative, *Y71A12B.17*. Like ZEEL-1_SOL_, the chimeric transgene, Y71A12B.17_TM_::ZEEL-1_SOL_, provided no rescue (Figure 7B). Assuming that this transgene was stably expressed, this result demonstrates that ZEEL-1_SOL_ cannot confer antidote activity to a related transmembrane domain. Additionally, this result demonstrates that the transmembrane domains of *zeel-1* and *Y71A12B.17* have diverged functionally since their common ancestor. The molecular basis of this divergence remains unclear, however, because the transmembrane domains of *zeel-1* and *Y71A12B.17* are only 35% identical at the amino acid level, with substitutions distributed throughout their length (Figure S7).

### Heat-Shock Expression of *peel-1* Kills Adult Animals, and Heat-Shock Expression of *zeel-1* Rescues This Lethality

**Figure 8:**
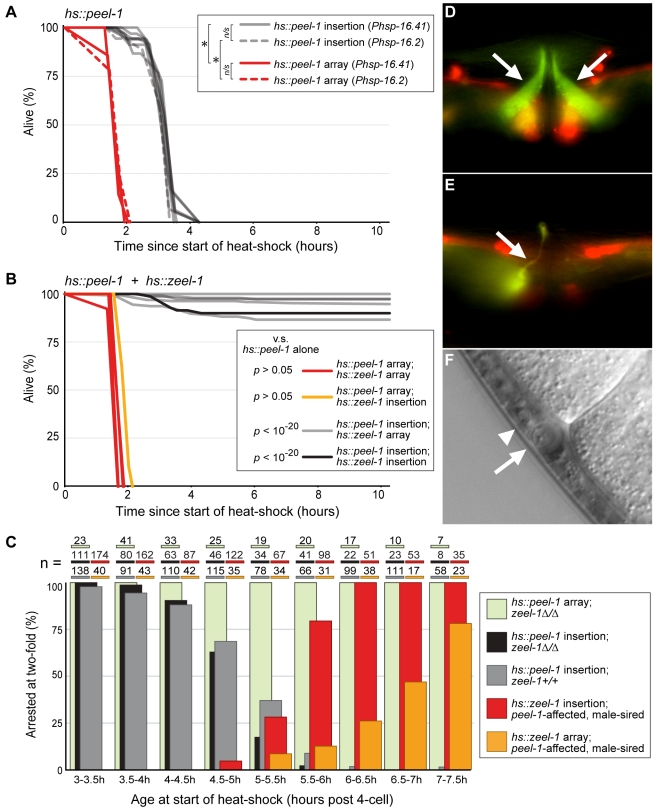
Ectopic expression of *peel-1* and *zeel-1* replicates *peel-1*-mediated toxicity and *zeel-1*-mediated rescue. (A) Survival curves for heat-shocked adult animals carrying extra-chromosomal arrays or single-copy genomic insertions of *Phsp-16.2::peel-1* or *Phsp-16.41::peel-1*. Time zero indicates the start of a 1-hour heat-shock at 34°C. Curves represent one array and one insertion of *Phsp-16.2::peel-1* and two independent arrays and seven independent insertions of *Phsp-16.41::peel-1*. Log-rank tests were used to compare (i) insertions versus arrays and (ii) *Phsp-16.2::peel-1* versus *Phsp-16.41::peel-1*. Data for independent arrays or insertions of *Phsp-16.41::peel-1* were combined prior to analysis. * *p*<10^−16^. *n/s*, *p*>0.05. *n* = 40–70 animals per curve. (B) One array and one insertion of *Phsp-16.41::peel-1* were chosen from (A) to be tested against five independent arrays and one single-copy insertion of *Phsp-16.41::zeel-1*. Animals carrying both types of transgenes were heat-shocked as in (A). Assays were truncated at 10 h post-heat-shock. Log-rank tests were used to compare each assay to the corresponding assay of *Phsp-16.41::peel-1* alone from (A) (*p* values shown). *n* = 45−180 animals per curve. (C) The following classes of embryos, aged 3 to 7.5 h post-four-cell stage, were heat-shocked for 20 min at 34°C: (i) *zeel-1(Δ)* embryos carrying a *Phsp-16.41::peel-1* array (pale green bars), (ii) *zeel-1(*Δ*)* and *zeel-1(+)* embryos carrying a *Phsp-16.41::peel-1* insertion (black and grey bars, respectively), and (iii) *peel-1*-affected, male-sired embryos carrying either an array or an insertion of *Phsp-16.41::zeel-1* (yellow and red bars, respectively). For each genotypic class, the proportion of embryos arresting at the 2-fold stage relative to all embryos that elongated to the 2-fold stage is plotted. Differences between the black and grey bars are not significant (*p*>0.05, χ^2^ tests for each age class). See Figure S8 for the full dataset. (D–E) Vulva regions of animals carrying an array of *Pexp-3::peel-1* and an integrated copy of *Pmyo-3::GFP*, a marker of the egg-laying muscles. Somatic inheritance of the *Pexp-3::peel-1* array was followed by co-injection markers, *Prab-3::mCherry* and *Pmyo-3::mCherry*, which express in neurons and the egg-laying and body-wall muscles, respectively. In (D), the egg-laying muscles (arrows) are morphologically normal and have not inherited the *Pexp-3::peel-1* array, as indicated by absence of *mCherry* expression. In (E), the left-hand egg-laying muscle is severely atrophied (arrow) and the right-hand egg-laying muscle is absent. The atrophied muscle cell expresses *mCherry*, indicating this cell has inherited the array of *Pexp-3::peel-1*. In both images, *mCherry* expression is also visible in body-wall muscles and neighboring neurons. (F) Dead GABA neuron (arrow) in an animal carrying an array of *Punc-47::peel-1*. The surrounding tissue, including a neighboring, non-GABA neuron (arrowhead), is morphologically normal.

To determine whether PEEL-1 can function as a toxin outside of embryos, we expressed *peel-1* ectopically in larvae and adults. *pee1-1* was expressed using each of two heat-shock promoters, *Phsp-16.2* and *Phsp-16.41* [35]. For each promoter construct, we generated both single-copy genomic insertions and extra-chromosomal arrays, which typically contain tens to hundreds of copies of a transgene [36]. Both types of animals grew normally under standard laboratory conditions, but a 1-h heat shock at 34°C was lethal to all: array-carrying adults died within 2 h after the start of heat-shock, and insertion-carrying animals within 4.5 h (Figure 8A). Faster killing of array-carrying animals is consistent with their higher *peel-1* dosage, and similar results were observed for heat-shocked larvae (unpublished data). In addition, aside from the gross phenotype of death, the heat-shocked animals showed defects in most, if not all, tissues. Beginning approximately 30 to 45 min before death, the body-wall and male-tail muscles hyper-contracted; vacuoles formed in many tissues (Figure S8K); the lumen of the excretory cell distended (Figure S8L); the gonad appeared to disintegrate (Figure S8M); and in hermaphrodites, the gonad and intestine occasionally exploded through the vulva. We conclude that PEEL-1 is a nearly universal toxin, affecting many developmental stages and cell types.

Next, we tested whether heat-shock expression of *zeel-1* could rescue the lethality caused by heat-shock expression of *peel-1*. We generated five extra-chromosomal arrays and one single-copy insertion of *Phsp-16.41::zeel-1*, and we tested these against one array and one insertion of *Phsp-16.41::peel-1*. Heat-shock expression of *zeel-1* was able to rescue the lethality caused by *Phsp-16.41::peel-1*, but only when *Phsp-16.41::peel-1* was expressed from insertions, not arrays (Figure 8B). The ability of *Phsp-16.41::zeel-1* to rescue *Phsp-16.41::peel-1* even when both were expressed from single-copy insertions (Figure 8B) indicates that insofar as these two transgenes produce equivalent levels of protein, *zeel-1*-mediated rescue does not require levels of ZEEL-1 to be higher than levels of PEEL-1.

### Sperm-Supplied PEEL-1 May Act Directly During the Two-Fold Stage

The fact that *peel-1*-affected embryos do not exhibit defects until late in development—at 2-fold stage—is surprising because sperm-supplied factors are thought to act only during egg-activation and first cleavage [16]. One explanation for this paradox is that the late-occurring defects might be a downstream manifestation of a cryptic defect earlier in development. Alternatively, sperm-supplied PEEL-1 might persist long after fertilization but only become toxic at the 2-fold stage. While we have been unable to visualize PEEL-1 after fertilization, using either PEEL-1::GFP or the anti-PEEL-1 antibody (presumably because PEEL-1 becomes too diffuse), three observations are consistent with PEEL-1 acting directly during the 2-fold stage.

First, pre-2-fold embryos were able to develop normally even when exposed to more PEEL-1 protein than is delivered by sperm. We heat-shocked pre-2-fold embryos, aged 3 to 7.5 h after the four-cell stage, carrying either an array or insertion of *Phsp-16.41::peel-1*. Embryos were heat-shocked for 20 min at 34°C. Longer and earlier heat-shock were not possible because even in wild-type embryos, such conditions cause premature arrest (personal observations). Except for occasional subtle shape defects during early elongation, more than 97% of array- and insertion-carrying embryos developed normally to the 2-fold stage (*n*≥200; Figure S8A). This result is consistent with sperm-supplied PEEL-1 being able to persist until the 2-fold stage without manifesting a visible defect earlier in development.

Second, heat-shock expression of *peel-1* as late as 30 min before the 2-fold stage phenocopied the defects observed in *peel-1*-affected embryos. In the heat-shocked embryos described above, embryos carrying an array of *Phsp-16.41::peel-1* uniformly arrested at the 2-fold stage and showed muscle detachment, epidermal leakage, and excretory cell distention (Figure 8C, Figure S8B–J). These defects were also observed among insertion-carrying embryos, although their occurrence required earlier induction of the transgene (Figure 8C). In addition, consistent with heat-shock treatment exposing embryos to more PEEL-1 protein than is delivered by sperm, the defects induced by heat-shock were often more severe than those observed in *peel-1*-affected embryos, and even among insertion-carrying embryos, these defects could not be rescued by endogenous expression of *zeel-1* (Figure 8C). These results demonstrate that as long as *peel-1* is expressed at or just before the 2-fold stage, presence of PEEL-1 in the early embryo is dispensable for the 2-fold arrest.

Finally, rescue of *peel-1*-affected embryos did not require early expression of *zeel-1*. We induced *zeel-1* expression in male-sired, *peel-1*-affected embryos by heat-shocking embryos carrying either an array or an insertion of *Phsp-16.41::zeel-1*. Heat-shock treatment rescued 53%–100% of array-carrying embryos (*n* = 17−43), as long as heat-shock treatment occurred at least 1 h before the 2-fold stage (Figure 8C). (Here rescue is defined as elongation past the 2-fold stage. See Figure S8A for the proportion of embryos that hatched.) Similar results were observed for insertion-carrying embryos, although rescue activity required earlier induction of the transgene (Figure 8C). These results imply that ZEEL-1 can neutralize sperm-supplied PEEL-1 at any time before the 2-fold stage. This scenario is temporally discordant with PEEL-1 causing a cryptic defect early in development.

### Cell-Specific Expression of *peel-1* Produces Cell-Specific Ablation

To test the cell-autonomy of *peel-1* killing, as well as the utility of *peel-1* as a tool for cell-specific ablation, we expressed *peel-1* under the control of each of two cell-specific promoters: *Punc-47*, which expresses in the GABA-ergic neurons [37], and *Pexp-3*, which expresses in the egg-laying muscles and the anal depressor muscle (C. Frøkjœr-Jensen, personal communication). For each promoter construct, we examined the presence or absence of the corresponding cell types for four independent extra-chromosomal arrays.

In both muscle cells and neurons, cell-specific expression of *peel-1* produced cell-specific ablation, although the efficacy of ablation varied among arrays. Three of the *Punc-47::peel-1* arrays killed 94.2%–99.8% (*n* = 241−453) of GABA-ergic neurons, although the fourth array killed only 28.6% (*n* = 350) of them. Each of the *Pexp-3::peel-1* arrays killed 6%–27% (*n* = 83−90) of anal depressor muscles and 74%–94% (*n* = 140−180) of egg-laying muscles. Of the egg-laying muscles that remained alive, all were severely atrophied (Figure 8E). Lower toxicity to the anal depressor muscle may have been caused by selection bias among our arrays, because animals lacking the anal depressor muscle were very severely constipated and therefore more slow-growing than others. In addition, even among animals in which the anal depressor remained alive, constipation was prevalent (Figure S9), indicating that function of this muscle was impaired.

Aside from the defects caused by ablation of the corresponding cell types, animals carrying either type of construct were morphologically and behaviorally normal, consistent with PEEL-1 acting cell-autonomously. In addition, with one exception, no defects were observed outside the ablated cells types (Figure 8F). The exception was that the three “high kill” *Punc-47::peel-1* arrays were lethal to the animal when inherited somatically along the lineage of the four RME neurons: For these three arrays, embryo and early larval lethality was very high (33.0%–57.7%; *n* = 327−1,095), and the only animals surviving to adulthood were those that lost the arrays somatically in the four RME neurons. Given that the RME neurons are not required for survival [38], this lethality implies that either (*i*) expression of *peel-1* in the RME neurons kills a neighboring cell nonautonomously or (*ii*) expression of *peel-1* is leaky and kills one or more essential cells along the RME lineage. While we did not distinguish between these possibilities, we note that the sister cell to one RME neuron is the excretory cell, which is essential for survival.

## Discussion

We have shown that the *peel-1/zeel-1* element in *C. elegans* is composed of two, tightly linked genes: a sperm-delivered toxin, *peel-1*, and an embryo-expressed antidote, *zeel-1*. *peel-1* and *zeel-1* are located adjacent to one another in the genome, and both genes encode transmembrane proteins. *peel-1* is expressed in the male germline, and its product is delivered to the embryo via fibrous body-membranous organelles. In the absence of *zeel-1*, sperm-supplied PEEL-1 causes dose-dependent, late-occurring defects in muscle and epidermal tissue. *zeel-1* is expressed transiently in the embryo, and tissue-specific expression of *zeel-1* produces tissue-specific rescue. The transmembrane domain of *zeel-1* is required and partially sufficient for function, and like *peel-1*, this domain is evolutionarily novel and does not occur outside *C. elegans*. Finally, although PEEL-1 and ZEEL-1 normally function in embryos, *peel-1* is lethal when expressed ectopically in adults, and this lethality is rescued by ectopic expression of *zeel-1*.

### Sperm and Early Embryos May Be Protected from PEEL-1

Given the evidence that sperm-supplied PEEL-1 may persist throughout embryogenesis and act directly during the 2-fold stage, PEEL-1 must be a remarkably potent toxin. Sperm are tiny in size compared to the oocyte, roughly 1% as large by volume [14], so PEEL-1 concentrations in the embryo are necessarily low. Moreover, assuming that PEEL-1 localizes to plasma membranes in the embryo, as might be expected for a FB-MO protein, then with each cell division, PEEL-1 will become more and more dilute relative to the total membrane component of the embryo.

Equivalently, the 2-fold stage of development must be remarkably sensitive to the toxic effects of PEEL-1. While the cause of this hypersensitivity remains unclear, we emphasize that the morphogenetic processes occurring at the 2-fold stage involve changes in cell shape and cell adhesion that are vastly more dramatic than those in earlier development. In addition, the two tissues most affected by PEEL-1—muscle and epidermis—are also the two tissues in which these morphogenetic changes are most pronounced.

The high potency of PEEL-1, combined with its widespread toxicity to a variety of cell types, highlights an unusual aspect of sperm cell biology: sperm are able to function normally, despite high concentrations of PEEL-1. While the mechanism of sperm protection remains unclear, we note that sperm differ from other cell types in three ways. First, sperm contain only a nucleus, some mitochondria, and FB-MOs; all other organelles and all ribosomes are excluded [39],[40]. Second, sperm lack an actin-based cytoskeleton [41], and instead crawl using polymers of the Major Sperm Protein [42]. Third, sperm sequester PEEL-1 in FB-MOs. Such sequestration is not possible in other cell types because FB-MOs are sperm-specific. In addition, although FB-MOs fuse with the plasma membrane upon sperm activation, they persist as permanent fusion pores [43]. This morphology prevents at least some FB-MO proteins from diffusing into the plasma membrane [44], and it may prevent diffusion of PEEL-1 as well.

The fact that pre-2-fold embryos are able to develop normally even when *peel-1* is induced by heat-shock indicates that pre-2-fold development is less sensitive than the 2-fold stage to the toxic effects of PEEL-1. However, given the hypersensitivity of the 2-fold stage, it remains unclear whether pre-2-fold embryos are fully resistant to PEEL-1 (like sperm), or whether PEEL-1 levels in the heat-shocked, pre-2-fold embryos were too low to produce general cytotoxic effects. While we cannot discount the possibility of full resistance, we note that in the heat-shocked embryos, the time interval between heat-shock and the 2-fold stage was 5 h, at most. Five hours was sufficient for necrosis to develop in heat-shocked adults, but the two heat-shock experiments are not directly comparable because the heat-shock response in adults and embryos may not be equivalent, and the duration of heat-shock was shorter in embryos than in adults.

### Possible Mechanisms of PEEL-1 Toxicity and ZEEL-1-Mediated Rescue

Because PEEL-1 has no sequence similarity to any other protein, the PEEL-1 sequence cannot be used to infer the mechanism of its toxicity. The fact that PEEL-1 is toxic even in extremely tiny amounts suggests that PEEL-1 might act catalytically—for example, by nucleating aggregation events or by acting as a transmembrane protease. The muscle hyper-contraction observed in heat-shocked adults, as well as the paralysis and 2-fold arrest observed in *peel-1*-affected embryos (which may in theory result from too much muscle contraction rather than too little), suggests that PEEL-1 might act by releasing intracellular calcium, perhaps by generating a membrane pore. It remains unclear, however, how calcium release alone can account for the epidermal defects observed in *peel-1*-affected embryos, because increased calcium signaling alone does not cause embryonic arrest [45] and is even known to suppress certain defects in epidermal morphogenesis [46]. In addition, it remains unclear how sperm might be protected from increased calcium, given the role of calcium in sperm activation [44].

Given the uncertain mechanism of PEEL-1 toxicity, there are also many possible mechanisms of ZEEL-1-mediated rescue. ZEEL-1 might promote degradation of PEEL-1, or it might prevent PEEL-1 from binding to its target, either by acting as a competitive inhibitor or by neutralizing PEEL-1 through direct interaction. While we have been unable to demonstrate a direct physical interaction between PEEL-1 and ZEEL-1, several observations are consistent with it. First, both PEEL-1 and ZEEL-1 are transmembrane proteins. ZEEL-1 localizes to cell membranes, and PEEL-1 localizes to FB-MOs. Assuming that FB-MOs do not endocytose during fertilization, localization to these organelles should deliver PEEL-1 to the plasma membrane of the zygote, where it should have the opportunity to encounter ZEEL-1 later in development. Second, the transmembrane domain of ZEEL-1 is required and partially sufficient for function, consistent with this domain binding directly to PEEL-1. Third, tissue-specific expression of *zeel-1* produces tissue-specific rescue, consistent with ZEEL-1 being able to neutralize PEEL-1 only when both proteins are present within the same cell. Fourth, ZEEL-1 can neutralize PEEL-1 toxicity even in adults, demonstrating that the genetic interaction between *peel-1* and *zeel-1* does not require any intermediaries specific to embryogenesis. Fifth, in both embryos and adults, ZEEL-1 is able to neutralize small but not large doses of PEEL-1. This dose-dependence implies that the genetic interaction between *peel-1* and *zeel-1* requires a minimum ratio of ZEEL-1 to PEEL-1.

### Comparison with Other Genetic Elements Causing Transmission Ratio Distortion

Like nearly all other “selfish” genetic elements whose genetic basis is known [2],[9],[47], the *peel-1/zeel-1* element experiences a suppression of recombination between component parts: The insertion/deletion of *peel-1* and *zeel-1* removes both genes at once, so the two genes cannot be separated by homologous recombination. This genomic organization has undoubtedly allowed *peel-1* to persist in spite of its toxic effects, because recombination breaking apart *peel-1* and *zeel-1* would have generated haplotypes carrying *peel-1* alone, and such haplotypes are effectively suicidal.

The *peel-1/zeel-1* element's mode of action is similar to that of *Wolbachia*, in that both types of elements act through paternal-effect killing [10],[11]. *Wolbachia*'s molecular mechanism is very different, however, because *Wolbachia* does not load sperm with an extra-nuclear toxin, but instead modifies the sperm pronucleus to undergo a chromatin condensation defect during the first mitotic division [48],[49]. In addition, *Wolbachia* is an intracellular bacterium, not a nuclear-encoded locus, and rescue of *Wolbachia*-mediated killing depends upon the contents of maternal ooplasm, not zygotic transcription of a nuclear-encoded gene. The *peel-1/zeel-1* element's mode of action is also similar to the maternal-effect killing and zygotic self-rescue of *Medea*-factors [50], although the extent of this similarity at the molecular level is unclear because the mechanism of *Medea*-factor killing is unknown [9].

### Sheltering of the *peel-1/zeel-1* Element by Near-Perpetual Homozygosity

Previously, we demonstrated that haplotypes carrying the *peel-1/zeel-1* element and haplotypes lacking it are maintained by balancing selection [13]. We hypothesized that the target of selection may be a linked polymorphism, rather than the *peel-1/zeel-1* element itself [13]. Under this scenario, *peel-1* may represent an unprecedented case of “inverted” sheltered load. Sheltered load refers to the incidental maintenance of deleterious alleles tightly linked to sites under balancing selection [51]. Ordinarily, sheltered load occurs when deleterious recessives arise on haplotypes maintained in persistent heterozygosity, such as those of major histocompatibility complex loci in vertebrates [52] or self-incompatibility loci in plants [53]. *peel-1* is like these deleterious recessives in that although *peel-1* has the potential to impose substantial genetic load on the species, its effects are rarely visible to natural selection. In the case of *peel-1*, however, sheltering is inverted because *peel-1* is only visible when heterozygous, and in *C. elegans*, heterozygosity is the exception rather than the norm.

Like any locus promoting its own transmission to the detriment of the rest of the genome, the *peel-1/zeel-1* element creates a selective environment favoring its own suppression. From a genic perspective, loci unlinked to the *peel-1/zeel-1* element suffer a fitness cost every time they are transmitted to a *peel-1*-affected embryo. As a consequence, mutations unlinked to *peel-1* and *zeel-1* that either suppress the activity of *peel-1* or mimic the activity of *zeel-1* will be favored by natural selection. Insofar as such alleles are accessible in mutational space, their absence further attests to the sheltering of the *peel-1/zeel-1* element by near-perpetual homozygosity. (The *peel-1* mutations in strains MY19 and EG4348 do not represent favored alleles because they do not arise on haplotypes suffering a fitness cost.)

### Evolutionary Origins of the *peel-1/zeel-1* Element

The insertion/deletion polymorphism of *peel-1* and *zeel-1* raises the following question: Did this indel polymorphism arise by an insertion event or by deletion of preexisting sequence? With respect to *zeel-1*, this polymorphism probably arose by a deletion event, because the divergence between *zeel-1* and its presumed ancestor, *Y71A12B.17*, predates allelic divergence at the *peel-1/zeel-1* locus. *zeel-1* and *Y71A12B.17* are 45% diverged at the amino acid level, and divergence at synonymous sites is saturated (see Materials and Methods). In comparison, the Bristol and Hawaii alleles of genes surrounding the indel polymorphism of *peel-1* and *zeel-1* are roughly 2% diverged at the amino acid level and 10%–16% diverged at synonymous sites [13], and this level of divergence is representative of the divergence between all haplotypes carrying the *peel-1/zeel-1* element and all haplotypes lacking it [13].

It is reasonable to suppose that the *peel-1*/*zeel-1* element originated as a weak toxin-antidote pair and then co-evolved into its current form. Yet given the low selective pressure for transmission ratio distortion in a self-fertilizing species, it is unlikely that *peel-1* and *zeel-1* co-evolved within *C. elegans* as result of this type of selective pressure alone. One possible solution to this paradox is that *peel-1* and *zeel-1* co-evolved in the out-crossing ancestor of *C. elegans*, where the selective pressure for transmission ratio distortion would have been much stronger. Another, non-mutually exclusive hypothesis is that *peel-1* was originally favored because it aided in another cellular process, such as sperm competition, and its toxicity to the embryo was initially mild and incidental. Under this scenario, *zeel-1* would have arisen to counteract the toxicity of *peel-1*, and once *zeel-1* became established, the presence of *zeel-1* would have allowed for stronger toxicity on the part of *peel-1* and, eventually, lethality in *zeel-1*'s absence. Regardless of the initial selective pressures favoring *peel-1* and *zeel-1*, however, the fact that both *peel-1* and the transmembrane domain of *zeel-1* are evolutionarily novel indicates that the self-promoting activity of the *peel-1*/*zeel-1* element arose fundamentally from the co-evolution of two novel proteins.

## Materials and Methods

### Strains

Strains were maintained at 19–23°C on NGM plates spotted with *E. coli* strain OP50. In all age-effect experiments, strains were strictly maintained at 20°C. For all transgenes described in this publication, only one array and/or one insertion is given in the strain list, although unless otherwise specified in the Results section, multiple independent arrays or insertions were examined.

CB4088: *him-5(e1490)* V.

CB4856: Hawaii natural isolate carrying *niDf9* I. *niDf9* designates the 19 kb deficiency spanning *peel-1* and *zeel-1*.

EG1285: *oxIs12[Punc-47::GFP; lin-15(+)] lin-15B(n765)* X.

EG4322: *ttTi5605* II*; unc-119(ed3)* III.

EG4348: Utah natural isolate carrying *peel-1(qq99)* I. EG4348 was collected by M. Ailion from Salt Lake City, Utah (this publication). *qq99* designates the naturally occurring nonsense mutation in *peel-1*.

EG5389: *qqIr7[peel-1(qq99)]* I; *oxIs494[Ppeel-1::GFP, Cbr-unc-119(+)]* II; *unc-119(ed3)* III.

EG5655: *qqIr7[peel-1(qq99)]* I; *oxSi19[peel-1(+), Cbr-unc-119(+)]* II; *unc-119(ed3)* III.

EG5766: *qqIr7[peel-1(qq99)]* I; *oxSi77[Ppeel-1::peel-1::GFP, Cbr-unc-119(+)]* II; *unc-119(ed3)* III.

EG5801: *oxSi87[Ppeel-1::peel-1_12a.a._::GFP, Cbr-unc-119(+)]* II; *unc-119(ed3)* III.

EG5955: *qqIr7[peel-1(qq99)]* I; *ttTi5605* II*; unc-119(ed3)* III; *oxEx1462[Phsp-16.41::peel-1, Cbr-unc-119(+), Pmyo-2::mCherry, Pmyo-3::mCherry, Prab-3::mCherry].*

EG5958: *qqIr7[peel-1(qq99)]* I; *oxSi186[Phsp-16.41::peel-1, Cbr-unc-119(+)]* II; *unc-119(ed3)* III.

EG5960: *qqIr7[peel-1(qq99)]* I; *oxSi188[Phsp-16.2::peel-1, Cbr-unc-119(+)]* II; *unc-119(ed3)* III.

EG5961: *qqIr7[peel-1(qq99)]* I; *ttTi5605* II*; unc-119(ed3)* III; *oxEx1464[Phsp-16.2::peel-1, Cbr-unc-119(+), Pmyo-2::mCherry, Pmyo-3::mCherry, Prab-3::mCherry]*.

EG6297: *qqIr5[niDf9]* I; *oxSi298[Phsp-16.41::zeel-1::tagRFP, Cbr-unc-119(+)]* II; *unc-119(ed3)* III.

EG6298: *qqIr5[niDf9]* I; *ttTi5605* II; unc*-119(ed3)* III; *oxEx1501[Phsp-16.41::zeel-1::tagRFP, Cbr-unc-119(+), Pmyo-2::GFP]*.

EG6301: *qqIr5[niDf9]* I; *ttTi5605* II*; unc-119(ed3)* III; *oxEx1504[Pexp-3::peel-1, Cbr-unc-119(+), Pmyo-2::mCherry, Pmyo-3::mCherry, Prab-3::mCherry*].

EG6306: *qqIr5[niDf9]* I; *ttTi5605* II*; unc-119(ed3)* III; *oxEx1509[Punc-47::peel-1, Cbr-unc-119(+), Prab-3::mCherry]*.

MT1344: *bli-3(e767) lin-17(n677)* I.

MT3301: *fem-1(hc17)* IV; *him-5(e1490)* V.

MY19: German natural isolate carrying *peel-1(qq98)* I. MY19 was collected from Roxel, Germany [54]. *qq98* designates the naturally occurring nonsense mutation in *peel-1*.

N2: Laboratory reference strain, Bristol.

PD4790: *mIs12[myo-2::GFP, pes-10::GFP* and *gut::GFP]*.

QX1015: *niDf9* I; *qqIr8[N2 = >CB4856, unc-119(ed3)]* III.

QX1197: *qqIr5[CB4856  = >N2, niDf9]* I. *qqIr5* is an 140–370 kb introgression from CB4856 into N2. This strain was used in some experiments instead of CB4856, in order to reduce the genetic variation segregating in the background.

QX1257: *niDf9* I; *qqIr8[unc-119(ed3)]* III; *qqIs2[zeel-1_genomic_::GFP, unc-119(+)*].

QX1264: *niDf9* I; *qqIr8[unc-119(ed3)]* III; *qqEx2[zeel-1_genomic_::GFP, unc-119(+)*].

QX1319: *zeel-1(tm3419)/hT2[qIs48]* I; *+/hT2[qIs48]* III.

QX1320: *qqIr6[EG4348 = >N2, peel-1(qq99)]* I; *unc-119(ed3)* III.

QX1384: *niDf9* I; *qqIr8[unc-119(ed3)]* III; *qqEx6[Pzeel-1:: zeel-1_SOL_, unc-119(+)]*.

QX1392: *qqIr6[peel-1(qq99)]* I; *unc-119(ed3)* III; *qqEx3[peel-1(+), unc-119(+)]*.

QX1409: *qqIr7[EG4348 = >N2, peel-1(qq99)]* I; *ttTi5605* II*; unc-119(ed3)* III.

QX1577: *qqIr5[niDf9]* I; *qqEx1[Pzeel-1::zeel-1_cDNA_::GFP, Pmyo-2::RFP]*.

QX1589: *qqIr5[niDf9]* I; *qqEx4[Pzeel-1::Y71A12B.17_TM_:: zeel-1_SOL_, Pmyo-2::RFP]*.

QX1605: *qqIr5[niDf9]* I; *ttTi5605* II*; unc-119(ed3)* III.

QX1607: *qqIr5[niDf9]* I; *qqEx5[Pzeel-1:: zeel-1_TM_, Pmyo-2::RFP]*.

QX1618: *qqIr5[niDf9]* I; *qqEx7[Plin-26::zeel-1, Pmyo-2::RFP]*.

QX1619: *qqIr5[niDf9]* I; *qqEx8[Phlh-1::zeel-1, Pmyo-2::RFP]*.

QX1624: *qqIr5[niDf9]* I; *oxSi186[Phsp-16.41::peel-1, Cbr-unc-119(+)]* II; *unc-119(ed3)* III.

QX1650: *oxSi19[peel-1(+), Cbr-unc-119(+)]* II.

QX1772: *qqIr5[niDf9]* I; *ttTi5605* II*; unc-119(ed3)* III; *oxEx1462[Phsp-16.41::peel-1, Cbr-unc-119(+), Pmyo-2::mCherry, Pmyo-3::mCherry, Prab-3::mCherry]*.

SJ4157: *zcIs21[Phsp-16::clpp-1(WT)::3xmyc-His tag+Pmyo-3::GFP]* V.

### Scoring Embryo Lethality

In all experiments except the age-effect experiment, embryo lethality from self-fertilizing hermaphrodites was scored by isolating hermaphrodites at the L4 stage and singling them to fresh plates the following day. After laying eggs for 8–10 h, the hermaphrodites were removed and embryos were counted. Unhatched embryos were counted ∼24 h later.

To score embryo lethality from mated hermaphrodites, three or four L4 hermaphrodites were mated to six to ten L4 or young adult males for 24–36 h. Hermaphrodites were then singled to fresh plates and embryo lethality was scored as above. Broods were examined for the presence of males 2–3 d later, and any broods lacking males were excluded. To allow male sperm to age within the reproductive tract of the hermaphrodite, mated hermaphrodites were removed from males, and lethality was scored among embryos laid 3 d after removal.

In the age-effect experiment, 91 *zeel-1(tm3419)peel-1(+)/niDf*9 hermaphrodites were singled at the L4 stage and transferred every 12 h to fresh plates. Hermaphrodites were discarded after the first 12-h period in which they failed to lay fertilized embryos. Total embryos were counted at the end of each laying period, and unhatched embryos were counted ∼24 h after each laying period had ended.

To score embryo lethality from partially mated hermaphrodites, 130 *zeel-1(tm3419)peel-1(+)/niDf*9 hermaphrodites were mated at the L4 stage to an equal number of PD4790 males, which carry an insertion of the fluorescent marker, *Pmyo-2::GFP*. After 24 h, hermaphrodites were singled and transferred every 12 h to fresh plates until day five. Embryo lethality was scored as above, except that after unhatched embryos were counted, hatched and unhatched progeny were classified as self- or cross-progeny according to presence of pharyngeal GFP. Hermaphrodites laying 100% self-progeny or more than 95% cross-progeny were excluded. The remaining hermaphrodites, which we define as “partially mated,” laid ∼10%–50% self-progeny. In these broods, we calculated the portion of self-progeny, laid during days 3 to 5, that failed to hatch.

### Mapping *peel-1* Mutations in MY19 and EG4348

Absence of the paternal-effect in EG4348 was mapped relative to *bli-3(e767)*, a visible marker located ∼10 cM from the *peel-1* interval. Mapping was performed as described [13]. Briefly, EG4348 males were crossed to MT1344 hermaphrodites, and F1 hermaphrodites were mated to CB4856 males. The resulting hermaphrodite progeny were allowed to self-fertilize, and their broods were scored for embryo lethality (i.e., presence of *peel-1* activity) and presence of Bli animals. Directionality with respect to *bli-3* could be inferred because *bli-3* is located at the left-hand tip of chromosome I.

Preliminary sequence analysis of the *peel-1* interval in EG4348 was performed by genotyping EG4348 with a subset of the markers listed in Table S2 of [13]. These markers tile across the *peel-1* interval, and they distinguish all haplotypes carrying an intact copy of the *peel-1/zeel-1* element from all haplotypes lacking it [13]. In other words, the Bristol-like alleles of these markers are in perfect linkage disequilibrium with presence of the *peel-1/zeel-1* element. At all markers we assayed, EG4348 carried the Bristol-like allele.

Fine-mapping in MY19 and EG4348 was performed by crossing each strain to MT1344 and collecting Lin Non-Bli and Bli Non-Lin recombinants in the F2 generation. Recombinant animals were genotyped (via a portion of their F3 broods) at each of two markers flanking the *peel-1* interval. The right-hand marker for the MY19 cross was a BstCI snip-SNP amplified with primers 5′-GTA TTC CGA CGA TTC GGA TG-3′ and 5′-CAT TGA GAA CAC AAA AAC AAA CG-3′. The right-hand marker for EG4348 cross was an AfeI snip-SNP amplified with primers 5′- GAC ATA TTT CCC GCA ACC TG-3′ and 5′- GTG ACG AGG CTT GAG GAT TC-3′. The left-hand marker for both crosses was a BanI snip-SNP amplified with primers 5′-CGC CAA ATA TGT TGT GCA GT-3′ and 5′-CAC CAC GTG TCC TTT CTC ATT-3′.

Recombinants breaking within the *peel-1* interval were homozygosed for the recombinant chromosome, and the resulting homozygotes were phenotyped for *peel-1* activity. Phenotyping was performed by crossing each line to CB4856 and scoring embryo lethality from self-fertilizing, F1 hermaphrodites, and from F1 males backcrossed to CB4856 hermaphrodites. Recombinants were classified as having *peel-1* activity if these crosses produced ∼25% and ∼50% embryo lethality, respectively. Next, the locations of recombination breakpoints were mapped more finely by sequencing six to ten sequence polymorphisms, located throughout the *peel-1* interval, that distinguish MY19 or EG4348 from Bristol. The MY19 polymorphisms were determined from the MY19 sequence described in [13], and the EG4348 polymorphisms were determined by amplifying and sequencing arbitrary fragments from this strain. Some polymorphisms are shared between MY19 and EG4348, and these were used in both crosses. For the most informative recombinants, we later sequenced across the entire breakpoint region in order to map these breakpoints to the level of adjacent polymorphisms. This approach mapped the *peel-1*-disrupting mutations to regions of 5 kb in MY19 and 8 kb in EG4348. These intervals were then sequenced in the corresponding strains, and all sequence polymorphisms were identified. Finally, we genotyped these polymorphisms in a panel of 38 wild strains previously identified as having intact *peel-1* activity [13]. These strains, as well as the primers used for genotyping them, are given in Figure S2. The MY19 sequence was deposited in GenBank previously [13], and the EG4348 sequence was deposited under accession number HQ291558.

### Identification of *peel-1* Transcript

RNA was collected from mixed-staged Bristol animals by freeze-cracking and extracting in Trizol (Invitrogen) according to the manufacturer's protocol. Reverse transcription-PCR (RT-PCR) was performed using pairs of primers flanking each candidate mutation in MY19 and EG4348. For each pair of primers, the forward and reverse primers were located ∼100 bp apart, and two reactions were performed, one using each of the two primers as the RT primer. Product was observed for only one pair of primers, and for that pair, only in one direction. These primers were 5′-ACA TGT ATC TTG ATC TGC CTG A-3′ (forward) and 5′-AAA AAT TAA CCA CAA TGA AGC AA-3′ (reverse), and product was only observed using the reverse primer as the RT primer. To recover the remainder of this putative transcript, 3′ and 5′ RACE were performed using standard methods [55]. For 3′ RACE, the RT reaction was performed using 5′-GTT TTC CCA GTC ACG ACT TTT TTT TTT TTT TTT TT-3′, and PCR was performed using the gene-specific primer, 5′-ACA TGT ATC TTG ATC TGC CTG A-3′ (forward), and the adaptor primer, 5′-GTT TTC CCA GTC ACG AC-3′ (reverse). For 5′ RACE, the RT reaction was performed using a gene-specific primer that spanned the putative stop codon, 5′-TCA ATT TCA TGG ATT TTC AAC A-3′, and PCR was performed using 5′-GGC CAC GCG TCG ACT AGT ACG GGI IGG GII GGG IIG-3′ (forward) and a nested, gene-specific primer, 5′-AAA AAT TAA CCA CAA TGA AGC AA-3′ (reverse). Then, a second round of PCR was performed using the adaptor primer, 5′-GGC CAC GCG TCG ACT AGT AC-3′ (forward), and another nested, gene-specific primer, 5′-AGA GCA ATA ACA TGC GCA AA-3′ (reverse). SuperScript III (Invitrogen) was used in all RT reactions, and PlatinumTaq (Invitrogen) was used for all PCR reactions. The *peel-1* transcript did not contain a splice leader sequence and was deposited in GenBank under accession number HQ291556. More recently, the *peel-1* transcript was identified independently by WormBase curators and assigned the identification number, *Y39G10AR.25*.

To search for transcripts carrying both *peel-1* and *zeel-1*, an RT reaction was performed using the *peel-1*-specific primer, 5′-AAA AAT TAA CCA CAA TGA AGC AA-3′, and PCR was performed using a forward primer located in the 3′ end of *zeel-1* (5′-CCA TCC GAG ATA ACC GAA AA-3′) and a reverse primer located in the 5′ end of *peel-1* (5′-AGA GCA ATA ACA TGC GCA AA-3′). No product was observed.

### Quantitative RT-PCR

CB4088 and MT3301 animals were grow at 15°C and synchronized at the L1 stage by bleaching and hatching overnight in M9. L1s were split into two populations, and one population was shifted to 25°C. When animals had reached young adulthood, hermaphrodites and males were separated by hand, and RNA was collected as above. Real-time PCR of *peel-1*, *spe-9*, and *rpl-26* was performed in triplicate, for 40 cycles, on an ABI 7900HT using the QuantiTect SYBR Green Kit (Qiagen). Relative expression levels of *peel-1* and *spe-9* were calculated separately for males and hermaphrodites, using the 2^−ΔΔCt^ method, with *rpl-26* as the endogenous control and the 15°C MT3301 sample as the reference sample. Primers used to amplify *peel-1* were 5′-TAC ACC CGT CAC ACC AAC TG-3′ and 5′-TCC GAC TAT GAT GTT CCA CAA-3′; primers for *spe-9* were 5′-CGG CTT GCA TAC ACA ATG AG-3′ and 5′-ACG CCA TGA CTC TTG CTC TT-3′; and primers for *rpl-26* were 5′-TCC AAT CAG AAC CGA TGA TG-3′ and 5′-GTG CAC AGT GGA TCC GTT AG-3′.

Among the hermaphrodite samples, relative expression levels of *peel-1* and *spe-9* were roughly equivalent, except for the 25°C MT3301 sample, where expression of *peel-1* and *spe-9* was undetectable. That is, in this sample, signal for *peel-1* and *spe-9* failed to rise above the detection threshold, even after 40 cycles, despite *rpl-26* amplifying normally.

### Single-Molecule FISH

Single molecule FISH of was performed as in [56], with the embryos and hermaphrodites squashed down to ∼9 µm thickness for imaging. Automated counting of nuclei in embryos was performed using software developed in [56],[57].

### Rescue of *peel-1*

Transgenic animals carrying *peel-1(+)* were generated by two methods: bombardment [58] and Mos1-mediated, single-copy insertion [59]. For bombardment, a fragment containing the Bristol allele of *peel-1*, along with ∼2.8 kb of upstream sequence and ∼1 kb of downstream sequence, was excised from fosmid WRM0633bE09 (Bioscience LifeSciences, Nottingham, UK) using AhdI and NgoMIV. This fragment was cloned into the yeast shuttle vector, pRS246 (ATCC, Manassas, VA), via yeast-mediated ligation [60] of the fragment's ends. The resulting plasmid, pHS11, was bombarded into QX1320, along with the *unc-119(+)* rescue vector, pDP#MM016B [61]. Bombardment was performed as in [62], although only extra-chromosomal arrays were recovered. Nine independent transgenic lines were tested for *peel-1* activity by crossing them to CB4856 and scoring embryo lethality from self-fertilizing, F1 hermaphrodites (self-cross) and F1 males backcrossed to CB4856 hermaphrodites (backcross).

For Mos1-mediated insertion, the *peel-1* fragment from pHS11 was amplified by PCR, using primers having NheI cut sites, and this amplicon was cut with NheI and ligated into pCFJ151 [59] linearized with AvrII. The resulting plasmid, pHS26, was injected into QX1409 along with the vectors needed to generate single-copy insertions [59]. Insertion-carrying animals were recovered by the direct insertion method [59], and five independent insertion-carrying lines were tested for *peel-1* activity as above. For one of the two insertions that did exhibit *peel-1* activity, the self-cross and backcross were repeated, and hatched progeny were collected and genotyped for a PCR-length polymorphism located less than 1 kb from *niDf9*. The primers used to amplify this polymorphism were 5′-TGG ATA CGA TTC GAG CTT CC-3′ (forward) and 5′-CCC CCT AAT TTC CAA GTG GT-3′ (reverse).

For three of the *peel-1* array lines, a small number of severely deformed L1s were observed in the backcross, similar to the “escapers” typically observed among *peel-1*-affected embryos sired by hermaphrodites. We suspected that these L1s had “escaped” the paternal-effect due to partial germline silencing of the *peel-1* arrays. Consistent with this hypothesis, we genotyped 13 of these animals, using the PCR-length polymorphism described above, and all were *zeel-1(niDf9)* homozygotes. We then calculated the frequency of these escapers relative to the total number of *peel-1*-affected progeny (i.e., relative to the total number of dead embryos and deformed L1s).

### *ZEEL-1::GFP* Fusion and Domain Swapping

*ZEEL-1::GFP* was generated by amplifying *GFP* from PD95.75 and inserting it into pHS4.1, a genomic subclone of *zeel-1(+)* described previously [13]. pHS4.1 was linearized with AhdI, and yeast-mediated ligation [60] was used to insert *GFP* just upstream of the *zeel-1* stop codon. Later, a second *ZEEL-1::GFP* construct was generated using the cDNA of *zeel-1*, instead of the genomic locus. This construct was generated by first cutting pHS4.1 with EcoNI and BglII, in order to remove the entire coding region of *zeel-1*, and then inserting a full cDNA of *zeel-1*, followed by *GFP*. The cDNA of *zeel-1* was cloned previously [13], and this replacement was performed using yeast-mediated ligation [60]. Both constructs showed full rescue of *peel-1*-affected embryos, and data from the two constructs were combined.

To generate *ZEEL-1_SOL_*, pHS4.1 was cut with EcoNI and KpnI, and the fragment containing *zeel-1* codons 5 to 205 was removed. The remaining fragment was then re-circularized, using yeast-mediated ligation [60], to fuse codon 4 to codon 206. To generate *ZEEL-1_TM_*, the entire coding region of *zeel-1* was excised from pHS4.1 using EcoNI and BglII, and this fragment was replaced with a partial cDNA of *zeel-1* encoding the first 205 amino acids of the protein. This replacement was performed using yeast-mediated ligation [60].

To generate *Y71A12B.17_TM_::ZEEL-1_SOL_*, the coding region of *zeel-1* was excised from pHS4.1, as above, and yeast mediated ligation [60] was used to replace this fragment with a partial cDNA of *Y71A12B.17*, followed by a partial cDNA of *zeel-1*. The resulting construct contained the *N*-terminal 207 codons of *Y71A12B.17* fused to the *C*-terminal 712 codons of *zeel-1*. The junction of this fusion was chosen to overlap a string of seven amino acids (KNERKEG) that are perfectly conserved between the two proteins. The *Y71A12B.17* cDNA was cloned by reverse transcribing RNA from the Bristol strain using primer 5′-TTG AAC AAA AAC AAT GGA TAT GTA A-3′, and then performing PCR using primers 5′-GGG GAC AAG TTT GTA CAA AAA AGC AGG CTT CAT GTC GGA TTT CGA CTC AGA-3′ (forward) and 5′-GGG GAC CAC TTT GTA CAA GAA AGC TGG GTC ATT TAT TAA CTC CAA CAA TGA TTC G-3′ (reverse). This PCR product was then cloned into the vector, pDONR221 (Invitrogen), using the Gateway cloning kit (Invitrogen). The *Y71A12B.17* cDNA differs slightly from the WormBase gene prediction and was deposited in GenBank under accession number HQ291557.

All constructs were bombarded [62] into QX1015 along with the *unc-119(+)* rescue vector, pDP#MM016B [61], or they were injected [63] into QX1197 at ∼80ng/µl, along with the fluorescent marker, *Pmyo-2::RFP* at 3 ng/µl. To test each transgene for its ability to rescue *peel-1*-affected embryos, transgenic animals were crossed to the Bristol strain, and lethality was scored among embryos derived from two crosses: self-fertilizing F1 hermaphrodites (self-cross) and F1 males backcrossed to hermaphrodites of the original transgenic line. To calculate hatch rates among *peel-1*-affected embryos inheriting *ZEEL-1::GFP* and *ZEEL-1_TM_*, transgenic animals were crossed to QX1319, and embryos were collected from (i) transgenic, self-fertilizing, F1, *zeel-1(tm3419)peel-1(+)/niDf9* hermaphrodites and (ii) transgenic, F1, *zeel-1(tm3419)peel-1(+)/niDf9* males backcrossed to non-transgenic, *niDf9/niDf9* hermaphrodites. Inheritance of *ZEEL-1::GFP* and *ZEEL-1_TM_* was inferred by expression of the co-injection marker, *Pmyo-2::RFP*, which can be scored even in arrested embryos.

### Other Transgenes

All other transgenes were generated using the three-site Gateway system from Invitrogen. This method allows three separate DNA fragments to be joined together and inserted into pCFJ150, which contains *Cbr-unc-119(+)* and the sequences needed for Mos1-mediated insertion at the *ttTi5605* Mos site on chromosome II [59]. In most cases, this method was used to join together a promoter of interest, a coding sequence, and a 3′ UTR.

### *Ppeel-1::GFP, Ppeel-1::PEEL-1_12a.a._::GFP*, and the *PEEL-1::GFP Fusion*

For *Ppeel-1::GFP*, we joined together the *peel-1* promoter, *GFP*, and the *peel-1* 3′UTR. For the *peel-1* promoter, we used all intergenic sequences between the *peel-1* start codon and last coding segment of *zeel-1* (i.e., 2,473 bp of sequence). The *peel-1* 3′ UTR was determined empirically and extended 86 bp downstream of the *peel-1* stop codon. For *GFP*, we used a variant containing S65C and three internal introns (identical to the variant in pPD95.75).

For *Ppeel-1::PEEL-1_12a.a._::GFP*, the PEEL-1 leader peptide was added by extending the promoter fragment to include the first 12 amino acids of PEEL-1. This signal peptide was discovered while we were investigating a possible regulatory role of the first intron of *peel-1*. We had generated a GFP reporter driven by the *peel-1* promoter and the first intron of *peel-1*, and this construct also happened to carry the first 12 amino acids of PEEL-1. GFP driven by this construct was packaged into sperm (unpublished data), and in order to confirm that sperm packaging was caused by the leader peptide, rather than the intron, we generated *Ppeel-1::PEEL-1_12a.a._::GFP* (which excludes the first intron). Conversely, we also generated a reporter carrying the first intron and a randomized leader peptide, and for this construct, no sperm packaging was observed (unpublished data).

To tag PEEL-1 with GFP, the promoter fragment was extended even further to include the entire *peel-1* gene, up to (but excluding) the stop codon.

All three constructs were injected into EG4322 or QX1409, and single-copy insertions were obtained using the direct insertion MosSCI method [59]. Three to six independent insertions were analyzed for each construct, and no differences were observed among insertions of the same construct. We note that although PEEL-1::GFP appears to localize normally, it failed to exhibit *peel-1* activity (unpublished data), presumably because the GFP tag inhibited function.

### *Plin-26::zeel-1 *and *Phlh-1::zeel-1*

For *Plin-26::zeel-1* and *Phlh-1::zeel-1*, the promoters of *lin-26* or *hlh-1* were joined to the cDNA of *zeel-1* and the 3′ UTR of *let-858*. For the promoters of *lin-26* and *hlh-1*, 7,122 bp of sequence and 3,037 bp of sequence upstream of the respective start codons were used. For the *let-858* 3′ UTR, 434 bp of sequence downstream of the stop codon was used. Transgenic animals were generated by injecting *Plin-26::zeel-1* and *Phlh-1::zeel-1* into QX1197 at ∼80 ng/µl, along with the fluorescent marker, *Pmyo-2::RFP* at 3 ng/µl.

To evaluate rescue among male-sired embryos, transgenic animals were crossed to QX1319, and transgenic, F1, *zeel-1(tm3419)peel-1(+)/niDf9* males were backcrossed to non-transgenic, *niDf9*/*niDf9* hermaphrodites. Embryos were dissected from these hermaphrodites and imaged every 10–20 min, starting before the 2-fold stage and ending at least 6 h after the 2-fold stage. To evaluate rescue among hermaphrodite-sired embryos, transgenic lines were crossed to QX1319, and embryo viability was scored among embryos collected from transgenic, self-fertilizing, F1, *zeel-1(tm3419)peel-1(+)/niDf9* hermaphrodites. Among both male- and hermaphrodite-sired embryos, inheritance of the transgene was inferred by expression of *Pmyo-2::RFP*.

### Heat-Shock Constructs and Constructs for Cell-Specific Expression of *peel-1*

*Phsp-16.41::peel-1*, *Phsp-16.2::peel-1*, *Pexp-3::peel-1*, and *Punc-47::peel-1* were generated by joining the *peel-1* cDNA downstream of the appropriate promoter and upstream of the *tbb-2* 3′ UTR. We describe the promoter and 3′UTR fragments in terms of length of sequence upstream or downstream of the appropriate start or stop codons: *Phsp-16.41* (501 bp), *Phsp-16.2* (493 bp), *Pexp-3* (2,877 bp), *Punc-47* (1,251 bp), and *tbb-2* 3′ UTR (331 bp). *Phsp-16.41::peel-1* and *Phsp-16.2::peel-1* were injected into QX1409 at 25 ng/µl, and arrays and MosSCI insertions were recovered as in [59]. The arrays carry co-injection markers *Pmyo-2::mCherry*, *Pmyo-3::mCherry*, and *Prab-3::mCherry*. *Pexp-3::peel-1* and *Punc-47::peel-1* were injected into QX1605 at 25 and 10 ng/µl, respectively, along with co-injection markers *Prab-3::mCherry*, *Pmyo-2::mCherry*, and *Pmyo-3::mCherry* (for *Pexp-3::peel-1*) and marker *Prab-3::mCherry* (for *Punc-47::peel-1*). In all cases, *Pmyo-3::mCherry* and *Prab-3::mCherry* were injected at 10 ng/µl, and *Pmyo-2::mCherry* was injected at 5 ng/µl.

*Phsp-16.41::zeel-1* was generated using the *hsp-16.41* promoter described above, the *zeel-1* cDNA, and the *let-858* 3′UTR fused downstream of *tagRFP*. *tagRFP* was added to confirm expression of *zeel-1* after heat-shock. *Phsp-16.41::zeel-1* was injected at 10 ng/µl into QX1605 and the arrays and the MosSCI insertion were recovered as in [59], except that a GFP-based co-injection marker (*Pmyo-2::GFP* injected at 2.5 ng/µl) was used in order to distinguish these arrays from the *Phsp-16.41::peel-1* arrays.

### Microscopy and Analysis of Live Embryos

Imaging of fixed embryos and live imaging of *ZEEL-1::GFP* embryos was performed on a PerkinElmer RS3 spinning disk confocal. All other imaging was performed on a Nikon 90i equipped with a CoolSNAP HQ2 camera and a X-Cite 120 Series fluorescent light source. Images were acquired and background subtracted with either Volocity (PerkinElmer) or NIS Elements (Nikon), and (in some cases) multiple channels were overlaid in Adobe Photoshop. To image dissected gonads, spermatocytes, and sperm, adult males or mated hermaphrodites were dissected into sperm media containing dextrose (50 mM Hepes, 1 mM MgSO_4_, 25 mM KCl, 45 mM NaCl, 5 mM CaCl_2_, 10 mM dextrose).

To measure the onset of epidermal leakage in *peel-1*-affected embryos, pre-arrest embryos were dissected from the following crosses. Hermaphrodite-sired embryos were dissected from (i) self-fertilizing, *zeel-1(tm3419)peel-1(+)/niDf*9 hermaphrodites and (ii) self-fertilizing, *zeel-1(tm3419)peel-1(+)/zeel-1(tm3419)peel-1(+)* hermaphrodites. Male-sired embryos were dissected from *niDf9/niDf9* hermaphrodites mated to three types of males: (i) *zeel-1(tm3419)peel-1(+)/niDf*9; (ii) *zeel-1(tm3419)peel-1(+)/zeel-1(+)peel-1(+)*; and (iii) *zeel-1(tm3419)peel-1(+)/zeel-1(+)peel-1(+)*; *oxSi19[peel-1(+)]/+.* The self-fertilizing hermaphrodites were aged 24 h post-L4 at the time of dissection, and mated hermaphrodites were aged 24–48 h at the time of dissection. After dissection, embryos were imaged every 10 min, starting before the 1.5-fold stage and ending 7 or more hours after the 1.5-fold stage. The onset of epidermal leakage was calculated as the time between the 1.5-fold stage and the first frame in which leakage was observed. Calculations were truncated at 7 h past the 1.5-fold stage because this represents 1 h after the average hatching time of wild-type embryos. Finally, embryos were binned into 30-min intervals in order to generate the inverted histograms shown in Figure 4A.

To calculate the percentage of *peel-1*-affected embryos elongating past 2-fold, *zeel-1(tm3419)peel-1(+)/niDf*9 hermaphrodites were isolated at the L4 stage and allowed to age for 24, 48, 60, and 72 h. Embryos were then dissected and imaged every 20 or 30 min for at least 10 h.

### Fixation of Sperm and Embryos

Anti-PEEL-1 is a rabbit polyclonal generated against the *C*-terminal 15 amino acids of PEEL-1. This antibody was generated and purified by GenScript, Piscataway, NJ. 1CB4 is a mouse monoclonal used to stain FB-MOs [64]. 1CB4 was a gift from Steven L'Hernault. To stain sperm, adult males were dissected into sperm media on charged slides, freeze-cracked in liquid nitrogen, and fixed overnight in −20°C methanol. Slides were washed with PBST (PBS+0.1% Triton-X 100), blocked for 30 min with PBST+0.5% BSA, and incubated for 4 h with anti-PEEL-1 (1/100) and 1CB4 (1/2,000), diluted in PBST+0.5% BSA. Slides were then washed three times in PBST and incubated for 2 h with Alexa568-labeled anti-mouse (1/500) and Alexa488-labeled anti-rabbit (1/500) (Invitrogen), diluted in PBST+0.5% BSA. Slides were washed again three times in PBST and mounted in Vectashield mounting media with DAPI.

To visualize actin filaments in *peel-1*-affected embryos, embryos were stained with Alexa568-labeled Phalloidin (Invitrogen) according to Protocol 7 in [65]. To visualize all the other proteins, embryos were stained with monoclonal antibodies MH2 (perlecan), DM5.6 (myosin heavy chain A), MH5 (VAB-10A), and MH4 (intermediate filaments). All monoclonals were obtained from The Developmental Studies Hybridoma Bank, Iowa City, Iowa. For these experiments, embryos were fixed for 10 min in 3% paraformaldehdye, freeze cracked in liquid nitrogen, and incubated for 5–10 min in −20°C methanol. Embryos were then washed three times in PBST and incubated overnight with the primary antibody diluted in PBST+1% BSA. MH2, MH4, and MH5 were diluted 1/150, and DM5.6 was diluted 1/1,000. Embryos were washed three times in PBST and incubated overnight with Alexa488-labeled anti-mouse (1/500) (Invitrogen), diluted in PBST+1% BSA. Embryos were washed again three times in PBST and mounted in Vectashield mounting media with DAPI.

### Phylogenetic Analysis of *zeel-1*

Separate phylogenetic trees were built for (i) *zyg-11* and all *zyg-11* homologs in *C. elegans* and (ii) *zyg-11* and all *zyg-11* homologs in *C. elegans*, *C. briggsae*, *C. remanei*, and *C. japonica*. *zyg-11* homologs were defined as all genes carrying the *zyg-11*-like leucine-rich repeat region. After removing the predicted transmembrane domains of ZEEL-1, Y71A12B.17, and Y55F3C.9, all protein sequences were aligned using MUSCLE [66]. The alignments were performed using the BLOSUM30 substitution matrix, a gap open penalty of −10, and a gap extend penalty of −1. The *C. elegans*–only alignment was trimmed to exclude residues having gaps in more than 90% of sequences, and the multi-species alignment was trimmed using the heuristic method, *automated1*, from TrimAL [67], which is optimized for maximum likelihood tree construction. Finally, phylogenetic trees were constructed using PhyML [68], using the LG substitution model [69], zero invariant sites, and four substitution rate categories. Branch support was determined using bootstrap sampling with 100 replicates.

Divergence between *zeel-1* and *Y71A12B.17* was determined by aligning the two proteins with MUSCLE [66], trimming the alignment of gaps, and using PAML [70] to calculate synonymous site divergence on the corresponding nucleotide sequences. (The total length of gaps was less than 0.1% of the length of the total alignment.) The summary statistics are as follows: number of synonymous sites = 715.5; number of non-synonymous sites = 2,002.5; synonymous substitutions per site (*d*_S_) = 1.0709; non-synonymous substitutions per site (*d*_N_) = 0.3267.

### Heat-Shock

Adults and larvae were heat-shocked by submerging sealed agar plates in a 34°C water bath for 1 h. Embryos were heat-shocked by mounting embryos on an agar pad, incubating the slide at 19–20°C for the prescribed number of hours before placing the slide on the floor of a sealed, 1 cm×8 cm×8 cm plastic box, and submerging the box in a 34°C water bath for 20 min. After heat-shock, embryos were imaged every 20 min for at least 10 h. Initially, embryos were staged directly by collecting and mounting four-cell embryos. Later, when it became clear that the vast majority of heat-shocked embryos developed to the 2-fold stage without defects or delay, throughput was increased by collecting mixed stage embryos and mounting, incubating, and heat-shocking as above. These embryos were staged relative to the time at which they initiated elongation and by comparing their morphology before heat-shock to images of embryos that had been staged using the direct method.

### Cell-Specific Killing

To quantify *peel-1*-mediated killing of the egg-laying muscles and the anal depressor muscle, the *Pexp-3::peel-1* arrays were crossed to SJ4157, which carries an integrated array of the muscle marker, *Pmyo-3::GFP*. In 1-d-old, F1 hermaphrodites, two of the four egg-laying muscles and the single anal depressor muscle were observed and classified as live or dead based on expression of *GFP*. Live egg-laying muscles were classified as morphologically normal or atrophied, and both live egg-laying muscles and live anal depressor muscles were then classified as mCherry^+^ or mCherry^−^, indicating that they had or had not inherited the *Pexp-3::peel-1* array. For each cell type, the percent of cells killed by the arrays was calculated assuming that all dead cells had inherited the array. In addition, each F1 animal was classified as constipated if it contained bacteria in the posterior intestine and as egg-laying defective if it contained 3-fold embryos in the uterus.

To quantify *peel-1*-mediated killing of GABA neurons, the *Punc-47::peel-1* arrays were crossed to EG1285, which carries an integrated array of the GABA-neuron marker, *Punc-47::GFP*. GABA-neurons were observed in F1 hermaphrodites, and *peel-1*-mediated killing was quantified as above. Typically, the DVB neuron and five to nine ventral cord neurons were scored per hermaphrodite. In addition, each animal was classified as mosaic or non-mosaic based on expression of the co-injection marker, *Prab-3::mCherry*.

## Supporting Information

Figure S1 *peel-1* and *zeel-1* are genetically separable. (A) Wild isolate EG4348 was crossed to Bristol and Hawaii, and lethality was scored among embryos collected from self-fertilizing F1 hermaphrodites and F1 males backcrossed to Hawaii hermaphrodites. A control cross, using F1 individuals derived from a cross between Bristol and Hawaii, was performed in parallel. (B) Embryo lethality was scored among embryos collected from three crosses: (i) self-fertilizing *zeel-1(tm3419)/zeel-1(+)peel-1(+)* hermaphrodites, (ii) self-fertilizing *zeel-1(tm3419)/zeel-1(*Δ*)peel-1(*Δ*)* hermaphrodites, and (iii) *zeel-1(tm3419)/zeel-1(*Δ*)peel-1(*Δ*)* males mated to *zeel-1(*Δ*)peel-1(*Δ*)/zeel-1(*Δ*)peel-1(*Δ*)* hermaphrodites. The allelic nature of *peel-1* on the haplotype carrying *zeel-1(tm3419)* is purposefully omitted because the goal of this experiment was to determine whether the deletion *tm3419* disrupts *peel-1* activity. Expected values were calculated under the hypothesis that *tm3419* creates a null allele of *zeel-1* but does not affect *peel-1*. Among embryos sired by hermaphrodites, slight decreases from 25% and 100% are expected because the paternal-effect killing is not fully penetrant when sperm derive from hermaphrodites [13]. (C) Absence of *peel-1* activity in EG4348 is genetically linked to *bli-3*, which is located on the left-hand tip of chromosome I, 10 cM from the *peel-1* interval. EG4348 was crossed to a strain of the Bristol background carrying *bli-3(e767)*, and F2 chromosomes were scored for *peel-1* activity and presence of the *bli-3(e767)* allele.
(TIF)

Figure S2 Some MY19 and EG4348 sequence changes are shared with wild isolates having intact *peel-1*. All of the sequence changes in MY19 and EG4348 located within the boxed intervals shown in Figure 1A were genotyped in a panel of 38 wild strains shown previously to have intact *peel-1* activity [13]. The position of each polymorphism (WormBase release May 2008 WS190/ce6) and the primers used to amplify and sequence it are listed diagonally above each column. Alleles unique to MY19 are shown in pink, alleles unique to EG4348 are shown in cyan, and alleles shared by at least one additional wild strain are shown in grey. Polymorphisms affecting the amino acid sequence of *peel-1* are indicated in the bottom row. *n/d*, not determined; (−), single base-pair deletion.
(TIF)

Figure S3 *peel-1* mRNA is not present in sperm. *peel-1* mRNAs were visualized in a wild-type, L4 hermaphrodite using single-molecule fluorescence in situ hybridization [56]. *peel-1* mRNAs are shown in red, and nuclei are stained with DAPI (cyan). Sperm and spermatocyte nuclei are labeled.
(TIF)

Figure S4 Age-related decrease in the lethality of *peel-1*-affected, hermaphrodite-sired embryos in 10 randomly selected broods. The results for 10 of the 91 broods used in the unmated experiment in Figure 5B are shown. Broods were selected using a random number generator. As described in Figure 5B, each hermaphrodite was followed from the onset of adulthood, and all embryos laid during the first 5 d of adulthood were collected. *n/a*, no embryos laid during this time period.
(TIF)

Figure S5 *ZEEL-1::GFP* is expressed transiently during embryogenesis. Time series images of a single embryo expressing *ZEEL-1::GFP* under the *zeel-1* promoter. Timeline indicates embryo age in hours post-four-cell stage.
(TIF)

Figure S6 Tissue-specific expression of *zeel-1* is partially sufficient for rescue of *peel-1*-affected, hermaphrodite-sired embryos. Embryo viability was calculated among *peel-1*-affected, hermaphrodite-sired embryos inheriting either *Plin-26::zeel-1* or *Phlh-1::zeel-1*. Embryos are grouped according to the age of the parent hermaphrodite. White bars indicate sibling controls that did not inherit the transgene. * *p*<10^−5^, χ^2^ tests.
(TIF)

Figure S7 The *zyg-11* family has expanded in *C. elegans*, *C. briggsae*, and *C. remanei*. (A) PhyML [68] was used to construct a maximum likelihood phylogeny of the protein sequences of all *zyg-11* homologs in *C. elegans*, *C. briggsae*, *C. remanei*, and *C. japonica*. As in Figure 7A, full-length protein sequences of all genes were used, except for the three proteins containing predicted transmembrane domains, ZEEL-1, Y71A12B.17, and Y55F3C.9. For these three proteins, highlighted with a shaded pink rectangle, the predicted transmembrane domains were excluded. Y71A12B.17 and the other proteins encoded by genes located in the tandem array are outlined with a pink dashed box. The frameshift in *Y71A12B.18* was corrected prior to analysis. Scale bar indicates amino acid substitutions per site. This value is highly deflated from its true value because the sequence alignment was heavily trimmed prior to constructing the phylogeny. Values on branches indicate percent bootstrap support. (B) Alignment of the amino acid sequences of the transmembrane domains of ZEEL-1 and Y71A12B.17. Sequences were aligned using MUSCLE [66], using default settings. Colors indicate amino acid classification: hydrophobic, including aromatic (black); acidic or basic (pink); and other (blue). Symbols below alignment indicate conservation. Above the alignment, horizontal bars indicate predicted transmembrane helices for ZEEL-1 (dark grey) and Y71A12B.17 (light grey). Predictions were generated using (from top to bottom): TopPred [71], Tmpred [72], TMHMM [73], SOSUI [74], PHDhtm [75], and HMMTOP [76].
(TIF)

Figure S8 Ectopic expression of *peel-1* and *zeel-1*. (A) The full dataset used to generate the plot in Figure 8C is shown. As described in Figure 8C, the following classes of embryos, aged 3 to 7.5 h post-four-cell stage, were heat-shocked for 20 min at 34°C: (i) wild-type; (ii) *zeel-1(*Δ*)/zeel-1(*Δ*)* embryos carrying a *Phsp-16.41::peel-1* array; (iii) *zeel-1(*Δ*)/zeel-1(*Δ*)* embryos carrying a *Phsp-16.41::peel-1* insertion; (iv) *zeel-1(+)/zeel-1(+)* embryos carrying a *Phsp-16.41::peel-1* insertion; (v) *peel-1*-affected, male-sired embryos carrying a *Phsp-16.41::zeel-1* array; and (vi) *peel-1*-affected, male-sired embryos carrying a *Phsp-16.41::zeel-1* insertion. Each embryo was classified as hatching (white) or arresting before the 2-fold stage (black), at the 2-fold stage (dark grey), or after the 2-fold stage (light grey). Numbers above bars indicate the total number of embryos in each age class. (B–J) Time series images of heat-shocked, *zeel-1(*Δ*)/zeel-1(*Δ*)* embryos carrying either an insertion (B–D) or an array (E–J) of *Phsp-16.41::peel-1*. When visible, epidermal leakage and excretory cell distention are labeled. In (G, J), tails are indicated to help orient the viewer. (K–M) Images of heat-shocked, adult hermaphrodites carrying an insertion of *Phsp-16.41::peel-1*. Animals were imaged shortly after paralysis had begun. Necrosis is visible in the head (K) and gonad (M), and the excretory cell is distended (L). (N–O) Images of animals carrying an array of *Pexp-3::peel-1* and an integrated copy of *Pmyo-3::GFP*, which serves as a marker of the anal depressor muscle. Intestinal bloating is visible in both animals, but only in (N) is the anal depressor muscle absent.
(TIF)

Figure S9 Cell-specific killing via ectopic expression of *peel-1*. (A–B) The *Pexp-3::peel-1* arrays were crossed to a strain carrying an insertion of *Pmyo-3::GFP*, which serves as a marker of the egg-laying muscles and the anal depressor muscle. Live muscle cells were classified as inheriting the array if they expressed the co-injection marker, *Pmyo-3::mCherry*. One hundred animals were scored for each array, and two of the four egg-laying muscles were scored per animal. (C) The *Punc-47::peel-1* arrays were crossed to a strain carrying an insertion of *Punc-47::GFP*, which serves as a marker for the GABA neurons. Live neurons were classified as inheriting the array if they expressed the co-injection marker, *Prab-3::mCherry*. For each array, 50–74 animals were scored, and 6 to 10 neurons were scored per animal.
(TIF)

Movie S1 Arrest phenotype of *peel-1*-affected embryos, focal plane 1. Time-lapse images of two *peel-1*-affected embryos are shown. Embryo on the right is approximately 2 h older than the embryo on the left. Images were captured every 10 min, beginning before elongation of both embryos and ending approximately 6 h after 2-fold arrest of the younger (left-hand) embryo.
(MOV)

Movie S2 Arrest phenotype of *peel-1*-affected embryos, focal plane 2. More proximal focal plane of the same *peel-1*-affected embryos shown in Movie S1. This focal plane highlights epidermal leakage in both embryos and thinning of the tail in the left-hand embryo.
(MOV)

Movie S3 Wild-type embryo. Time-lapse images of a wild-type embryo. This embryo is approximately 30 min older than the left-hand embryo in Movies S1–S2. Images were captured every 10 min, beginning approximately 4 h before the 2-fold stage and ending at hatching, which occurs approximately 6 h after the 2-fold stage.
(MOV)

## Abbreviations

FB-MO: fibrous body-membranous organelle
FISH: fluorescence in situ hybridization
RT-PCR: reverse transcription polymerase chain reaction
RACE: rapid amplification of cDNA ends
