# Supplementary figures and images for: A Novel Sperm-Delivered Toxin Causes Late-Stage Embryo Lethality and Transmission Ratio Distortion in C. elegans
Source: PLoS Biol. 2011 Jul 26;9(7):e1001115. doi: 10.1371/journal.pbio.1001115 (PMC3144186; doi:10.1371/journal.pbio.1001115)

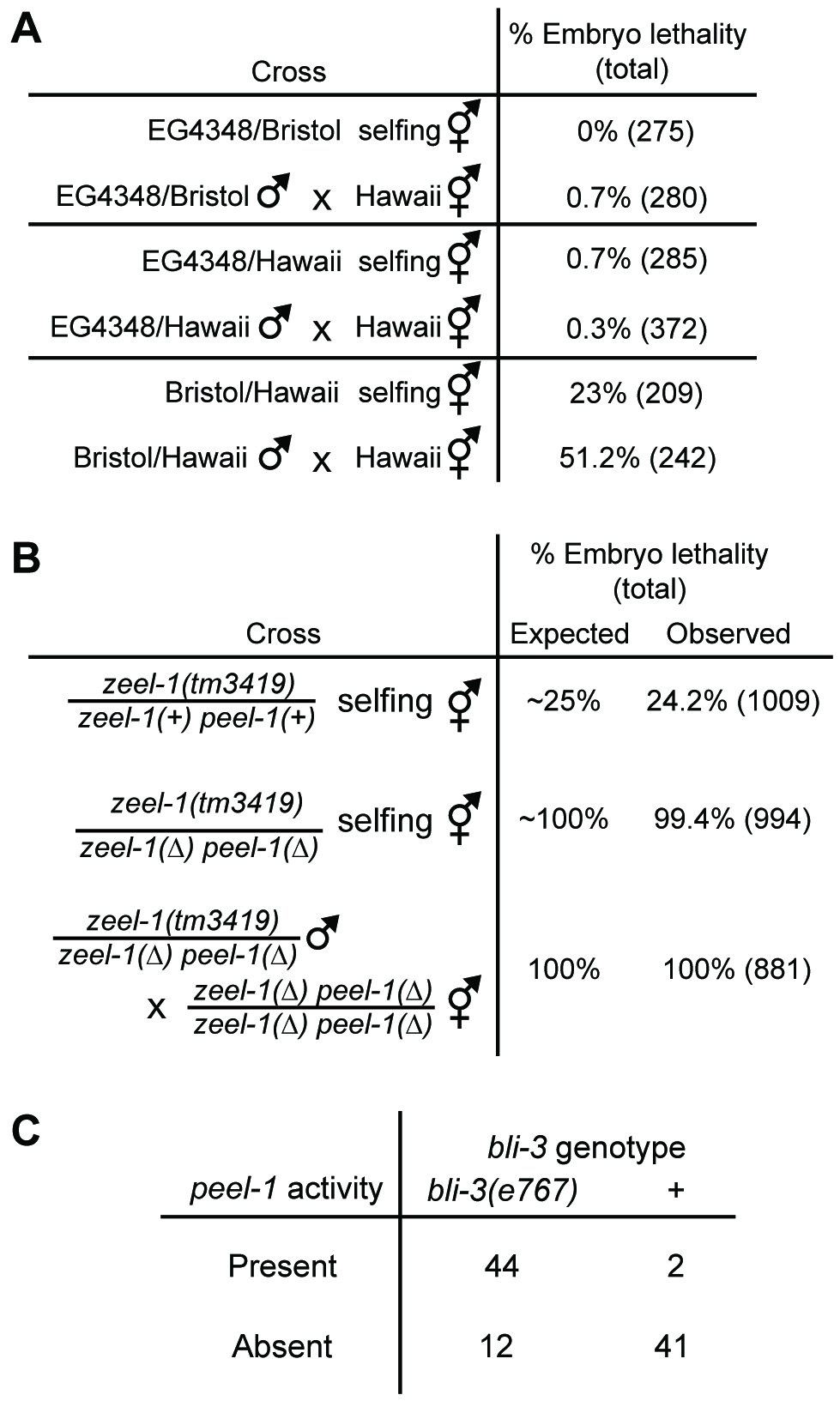

Supplement: Figure S1 — peel-1 and zeel-1 are genetically separable. (A) Wild isolate EG4348 was crossed to Bristol and Hawaii, and lethality was scored among embryos collected from self-fertilizing F1 hermaphrodites and F1 males backcrossed to Hawaii hermaphrodites. A control cross, using F1 individuals derived from a cross between Bristol and Hawaii, was performed in parallel. (B) Embryo lethality was scored among embryos collected from three crosses: (i) self-fertilizing zeel-1(tm3419)/zeel-1(+)peel-1(+) hermaphrodites, (ii) self-fertilizing zeel-1(tm3419)/zeel-1(Δ)peel-1(Δ) hermaphrodites, and (iii) zeel-1(tm3419)/zeel-1(Δ)peel-1(Δ) males mated to zeel-1(Δ)peel-1(Δ)/zeel-1(Δ)peel-1(Δ) hermaphrodites. The allelic nature of peel-1 on the haplotype carrying zeel-1(tm3419) is purposefully omitted because the goal of this experiment was to determine whether the deletion tm3419 disrupts peel-1 activity. Expected values were calculated under the hypothesis that tm3419 creates a null allele of zeel-1 but does not affect peel-1. Among embryos sired by hermaphrodites, slight decreases from 25% and 100% are expected because the paternal-effect killing is not fully penetrant when sperm derive from hermaphrodites [13]. (C) Absence of peel-1 activity in EG4348 is genetically linked to bli-3, which is located on the left-hand tip of chromosome I, 10 cM from the peel-1 interval. EG4348 was crossed to a strain of the Bristol background carrying bli-3(e767), and F2 chromosomes were scored for peel-1 activity and presence of the bli-3(e767) allele. (TIF) [file pbio.1001115.s001.tif]

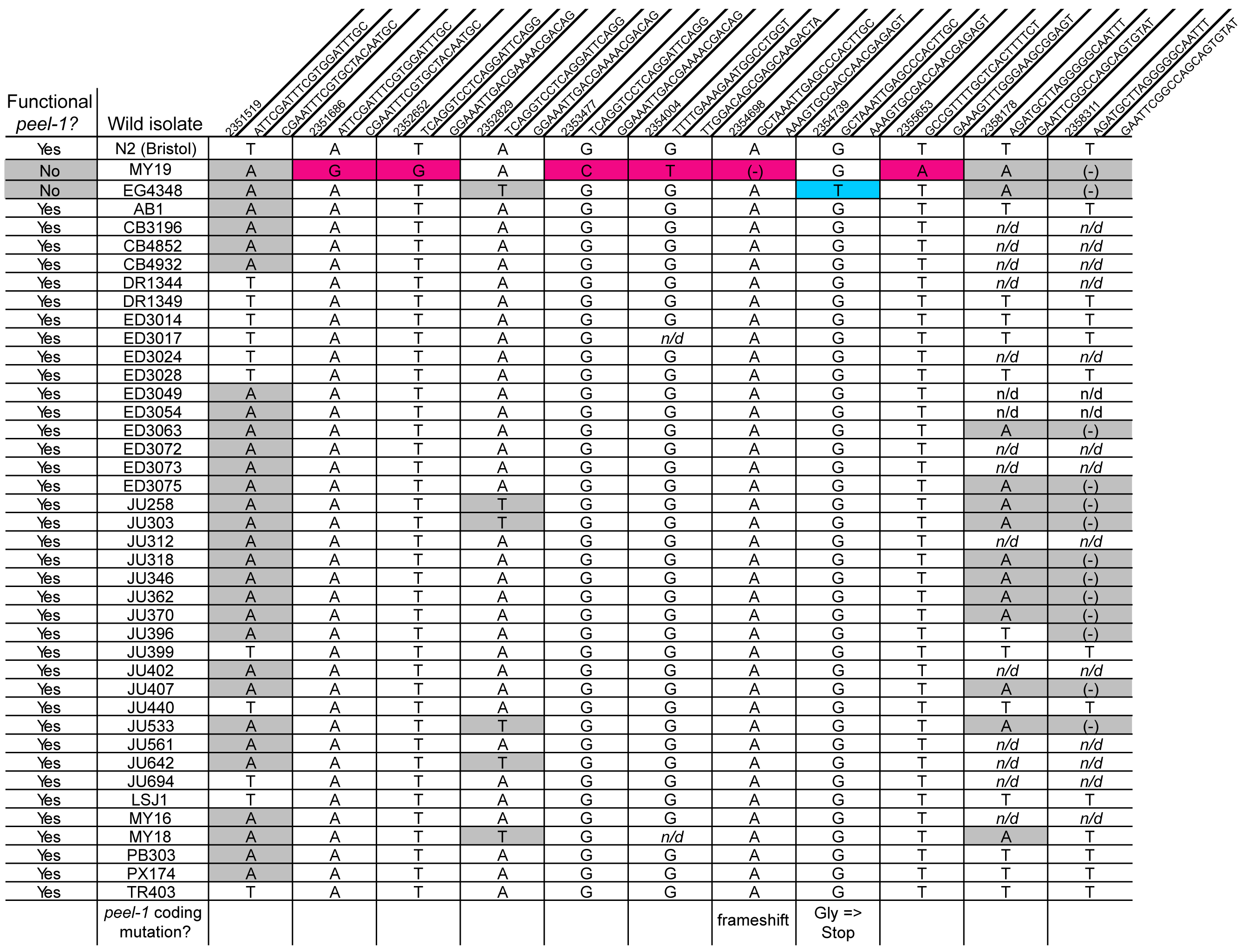

Supplement: Figure S2 — Some MY19 and EG4348 sequence changes are shared with wild isolates having intact peel-1. All of the sequence changes in MY19 and EG4348 located within the boxed intervals shown in Figure 1A were genotyped in a panel of 38 wild strains shown previously to have intact peel-1 activity [13]. The position of each polymorphism (WormBase release May 2008 WS190/ce6) and the primers used to amplify and sequence it are listed diagonally above each column. Alleles unique to MY19 are shown in pink, alleles unique to EG4348 are shown in cyan, and alleles shared by at least one additional wild strain are shown in grey. Polymorphisms affecting the amino acid sequence of peel-1 are indicated in the bottom row. n/d, not determined; (−), single base-pair deletion. (TIF) [file pbio.1001115.s002.tif]

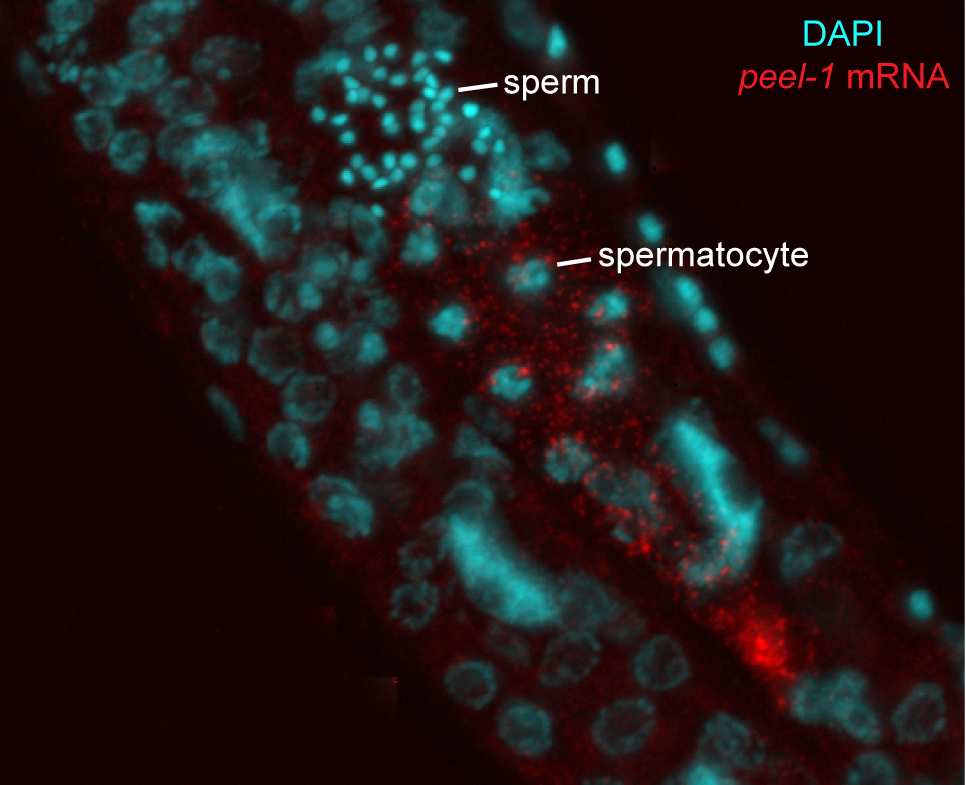

Supplement: Figure S3 — peel-1 mRNA is not present in sperm. peel-1 mRNAs were visualized in a wild-type, L4 hermaphrodite using single-molecule fluorescence in situ hybridization [56]. peel-1 mRNAs are shown in red, and nuclei are stained with DAPI (cyan). Sperm and spermatocyte nuclei are labeled. (TIF) [file pbio.1001115.s003.tif]

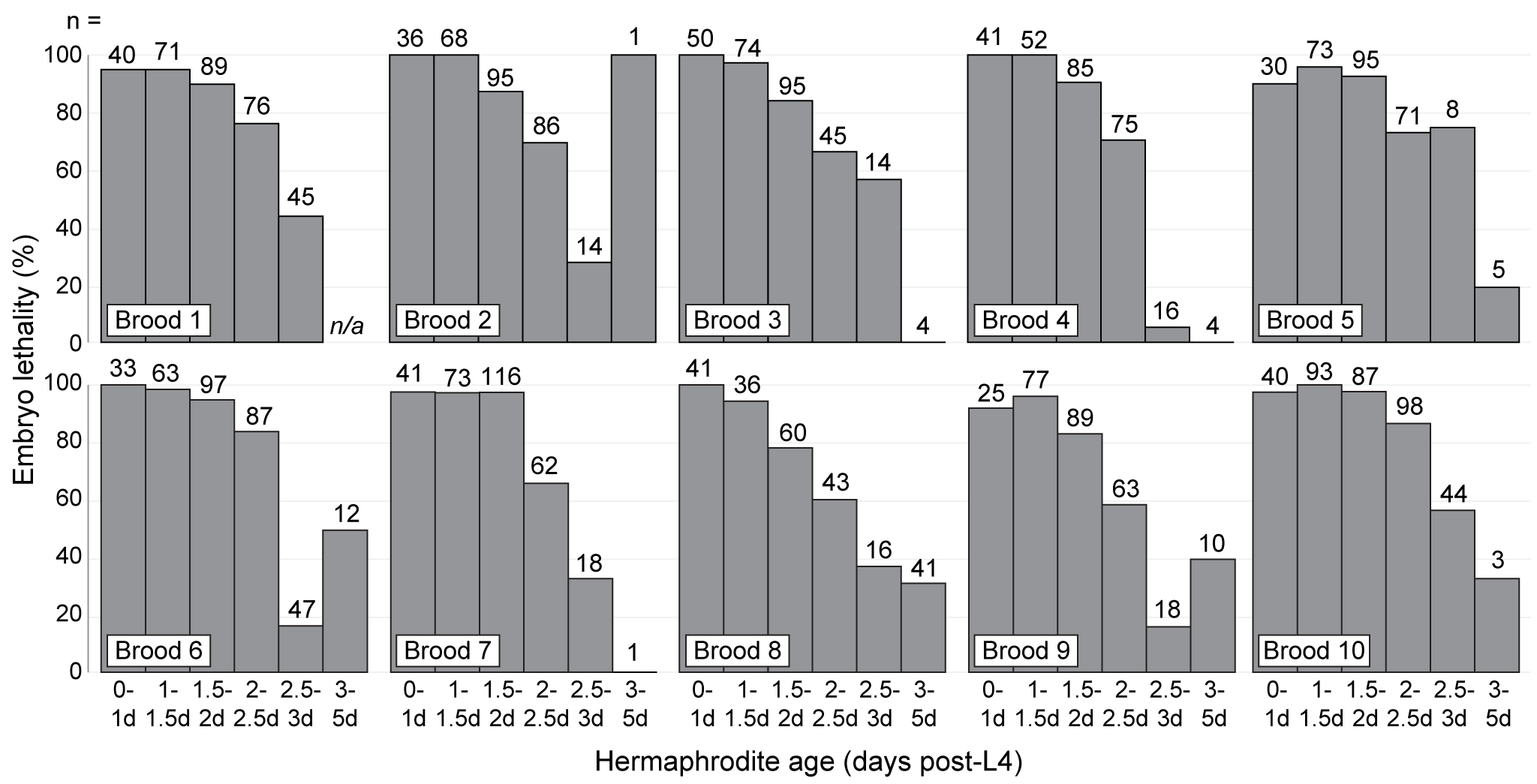

Supplement: Figure S4 — Age-related decrease in the lethality of peel-1-affected, hermaphrodite-sired embryos in 10 randomly selected broods. The results for 10 of the 91 broods used in the unmated experiment in Figure 5B are shown. Broods were selected using a random number generator. As described in Figure 5B, each hermaphrodite was followed from the onset of adulthood, and all embryos laid during the first 5 d of adulthood were collected. n/a, no embryos laid during this time period. (TIF) [file pbio.1001115.s004.tif]

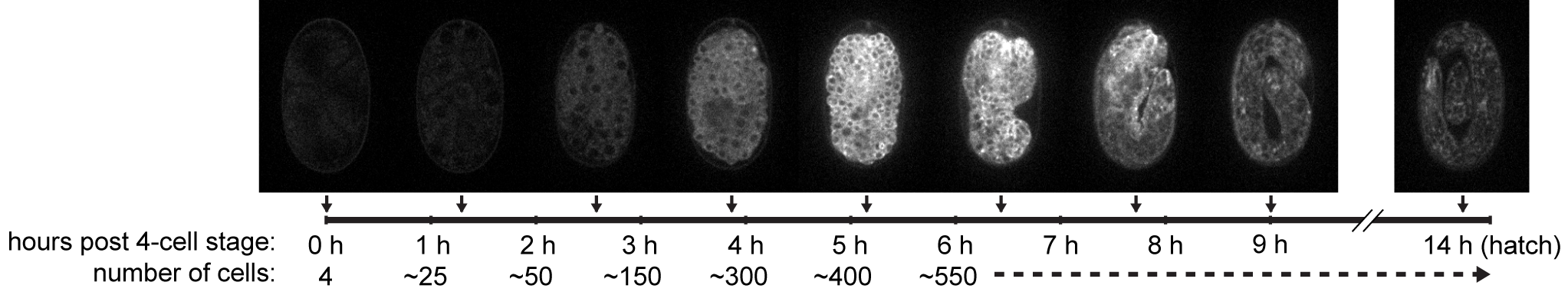

Supplement: Figure S5 — ZEEL-1::GFP is expressed transiently during embryogenesis. Time series images of a single embryo expressing ZEEL-1::GFP under the zeel-1 promoter. Timeline indicates embryo age in hours post-four-cell stage. (TIF) [file pbio.1001115.s005.tif]

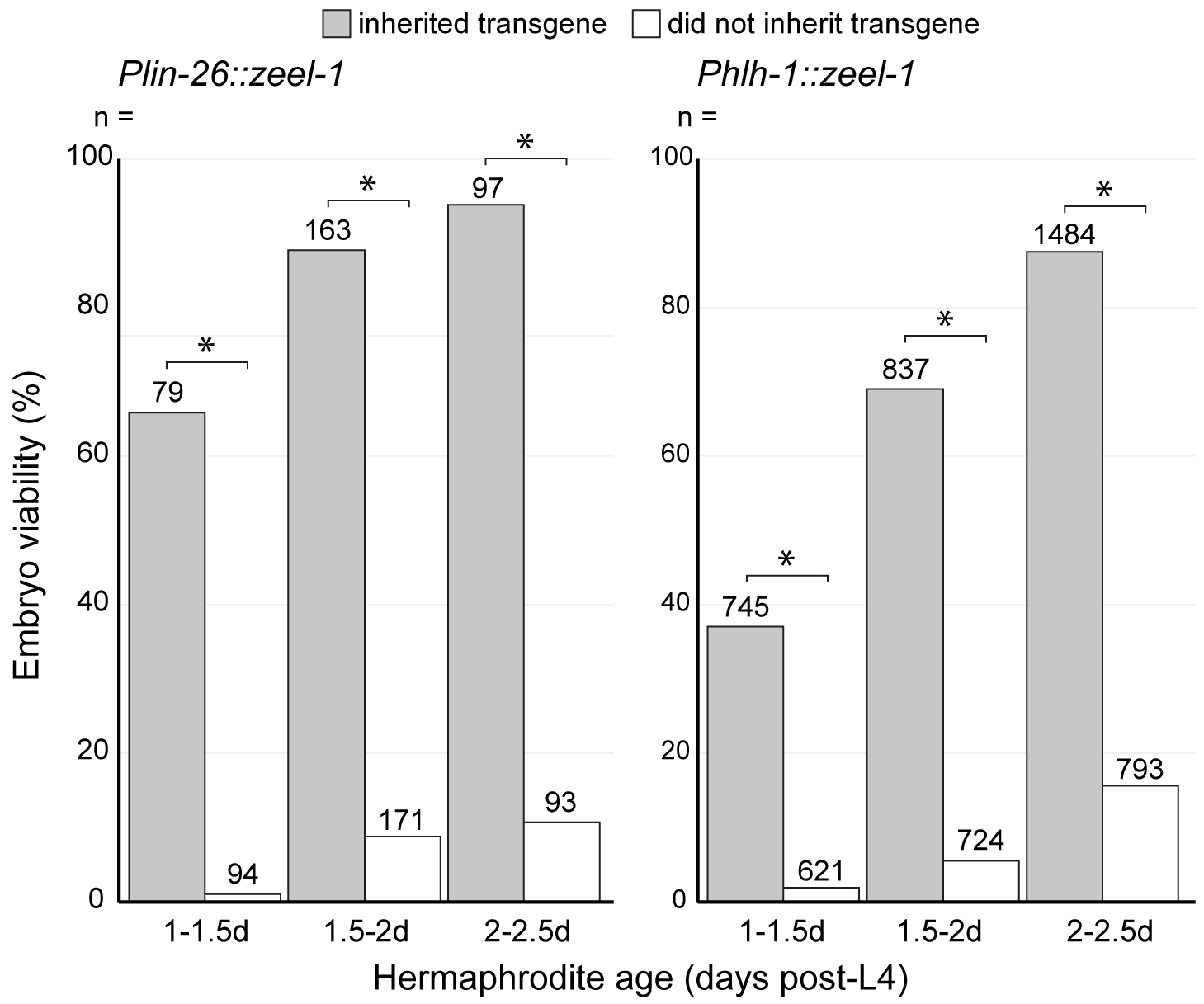

Supplement: Figure S6 — Tissue-specific expression of zeel-1 is partially sufficient for rescue of peel-1-affected, hermaphrodite-sired embryos. Embryo viability was calculated among peel-1-affected, hermaphrodite-sired embryos inheriting either Plin-26::zeel-1 or Phlh-1::zeel-1. Embryos are grouped according to the age of the parent hermaphrodite. White bars indicate sibling controls that did not inherit the transgene. * p<10−5, χ2 tests. (TIF) [file pbio.1001115.s006.tif]

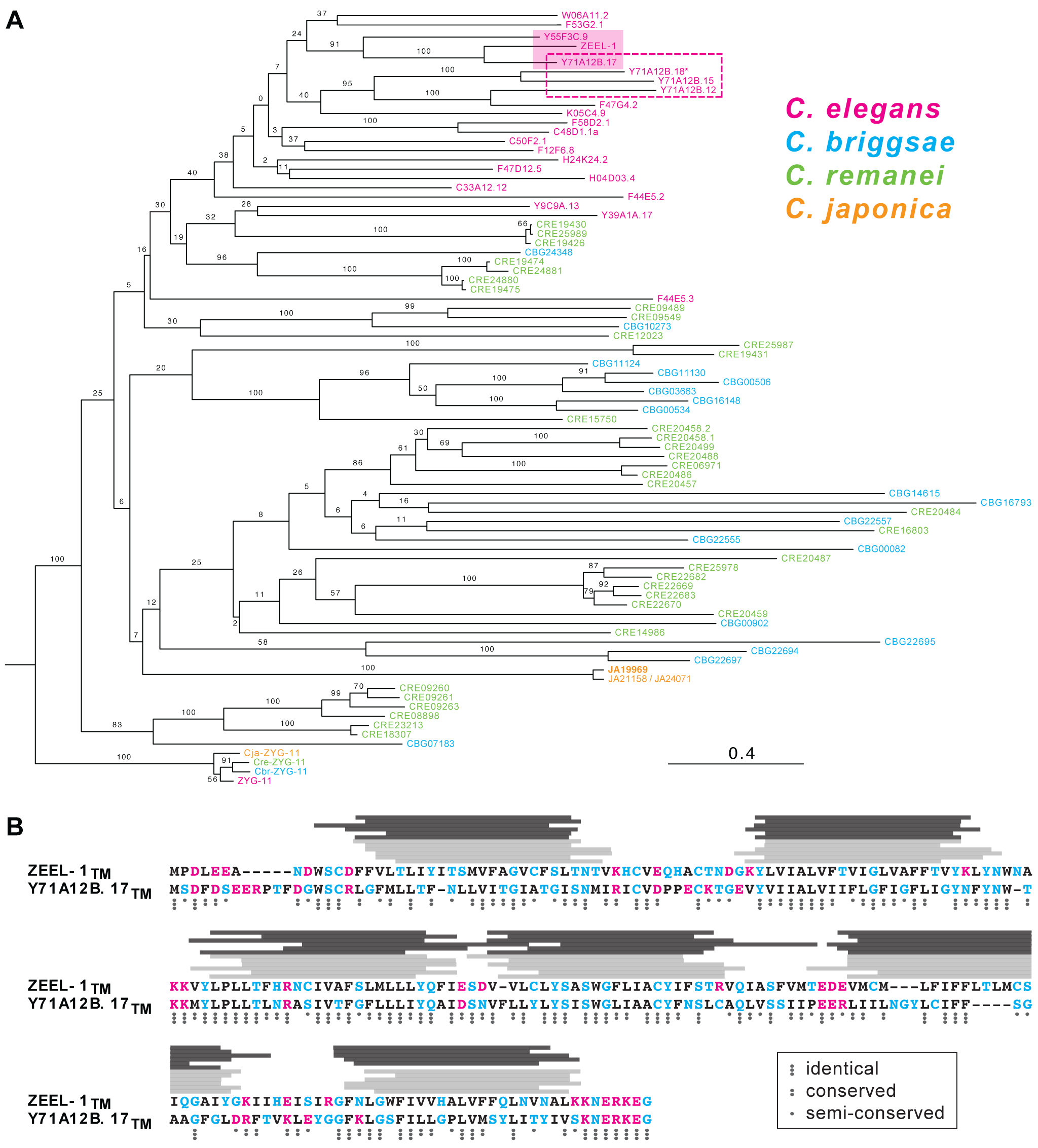

Supplement: Figure S7 — The zyg-11 family has expanded in C. elegans, C. briggsae, and C. remanei. (A) PhyML [68] was used to construct a maximum likelihood phylogeny of the protein sequences of all zyg-11 homologs in C. elegans, C. briggsae, C. remanei, and C. japonica. As in Figure 7A, full-length protein sequences of all genes were used, except for the three proteins containing predicted transmembrane domains, ZEEL-1, Y71A12B.17, and Y55F3C.9. For these three proteins, highlighted with a shaded pink rectangle, the predicted transmembrane domains were excluded. Y71A12B.17 and the other proteins encoded by genes located in the tandem array are outlined with a pink dashed box. The frameshift in Y71A12B.18 was corrected prior to analysis. Scale bar indicates amino acid substitutions per site. This value is highly deflated from its true value because the sequence alignment was heavily trimmed prior to constructing the phylogeny. Values on branches indicate percent bootstrap support. (B) Alignment of the amino acid sequences of the transmembrane domains of ZEEL-1 and Y71A12B.17. Sequences were aligned using MUSCLE [66], using default settings. Colors indicate amino acid classification: hydrophobic, including aromatic (black); acidic or basic (pink); and other (blue). Symbols below alignment indicate conservation. Above the alignment, horizontal bars indicate predicted transmembrane helices for ZEEL-1 (dark grey) and Y71A12B.17 (light grey). Predictions were generated using (from top to bottom): TopPred [71], Tmpred [72], TMHMM [73], SOSUI [74], PHDhtm [75], and HMMTOP [76]. (TIF) [file pbio.1001115.s007.tif]

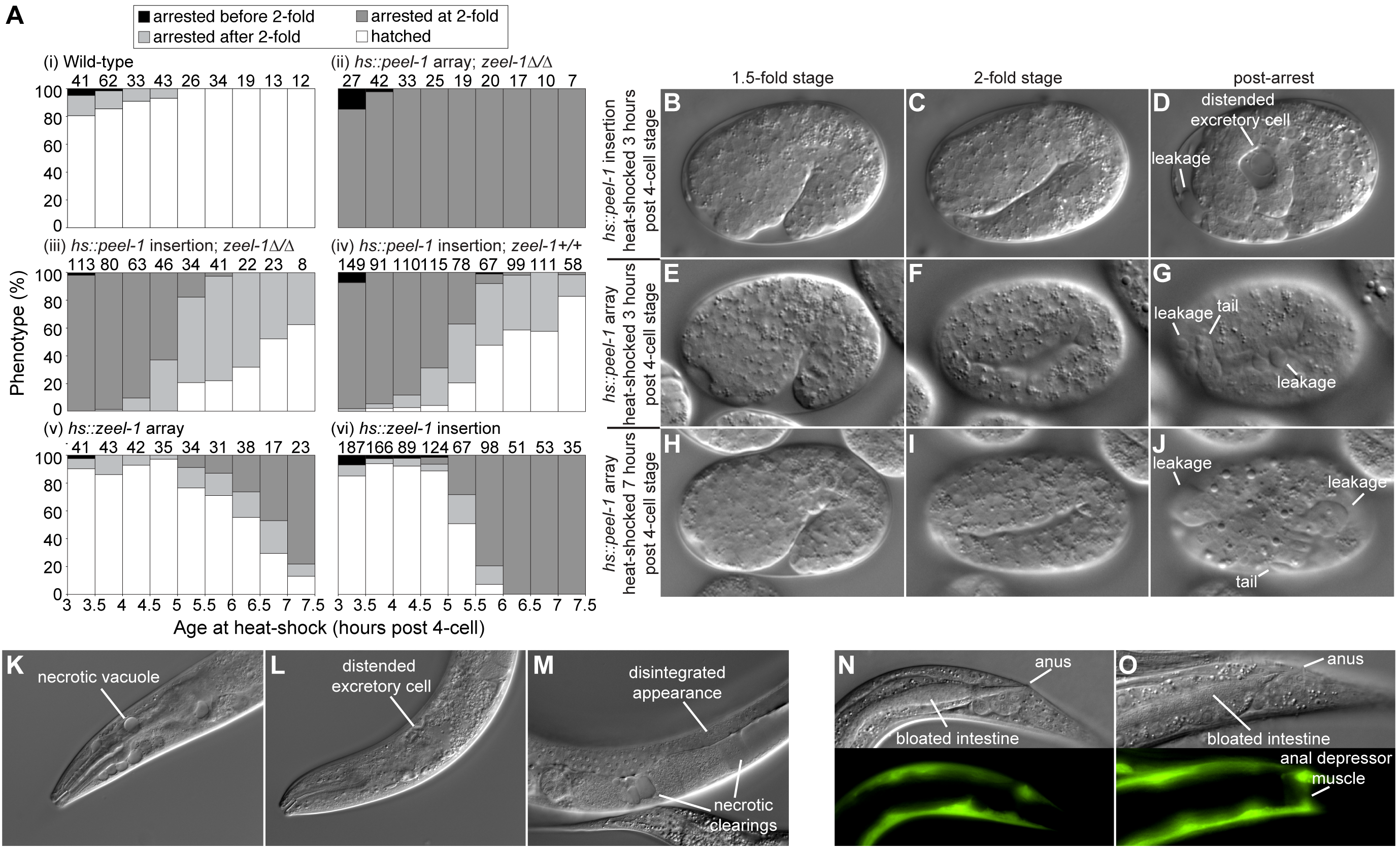

Supplement: Figure S8 — Ectopic expression of peel-1 and zeel-1. (A) The full dataset used to generate the plot in Figure 8C is shown. As described in Figure 8C, the following classes of embryos, aged 3 to 7.5 h post-four-cell stage, were heat-shocked for 20 min at 34°C: (i) wild-type; (ii) zeel-1(Δ)/zeel-1(Δ) embryos carrying a Phsp-16.41::peel-1 array; (iii) zeel-1(Δ)/zeel-1(Δ) embryos carrying a Phsp-16.41::peel-1 insertion; (iv) zeel-1(+)/zeel-1(+) embryos carrying a Phsp-16.41::peel-1 insertion; (v) peel-1-affected, male-sired embryos carrying a Phsp-16.41::zeel-1 array; and (vi) peel-1-affected, male-sired embryos carrying a Phsp-16.41::zeel-1 insertion. Each embryo was classified as hatching (white) or arresting before the 2-fold stage (black), at the 2-fold stage (dark grey), or after the 2-fold stage (light grey). Numbers above bars indicate the total number of embryos in each age class. (B–J) Time series images of heat-shocked, zeel-1(Δ)/zeel-1(Δ) embryos carrying either an insertion (B–D) or an array (E–J) of Phsp-16.41::peel-1. When visible, epidermal leakage and excretory cell distention are labeled. In (G, J), tails are indicated to help orient the viewer. (K–M) Images of heat-shocked, adult hermaphrodites carrying an insertion of Phsp-16.41::peel-1. Animals were imaged shortly after paralysis had begun. Necrosis is visible in the head (K) and gonad (M), and the excretory cell is distended (L). (N–O) Images of animals carrying an array of Pexp-3::peel-1 and an integrated copy of Pmyo-3::GFP, which serves as a marker of the anal depressor muscle. Intestinal bloating is visible in both animals, but only in (N) is the anal depressor muscle absent. (TIF) [file pbio.1001115.s008.tif]

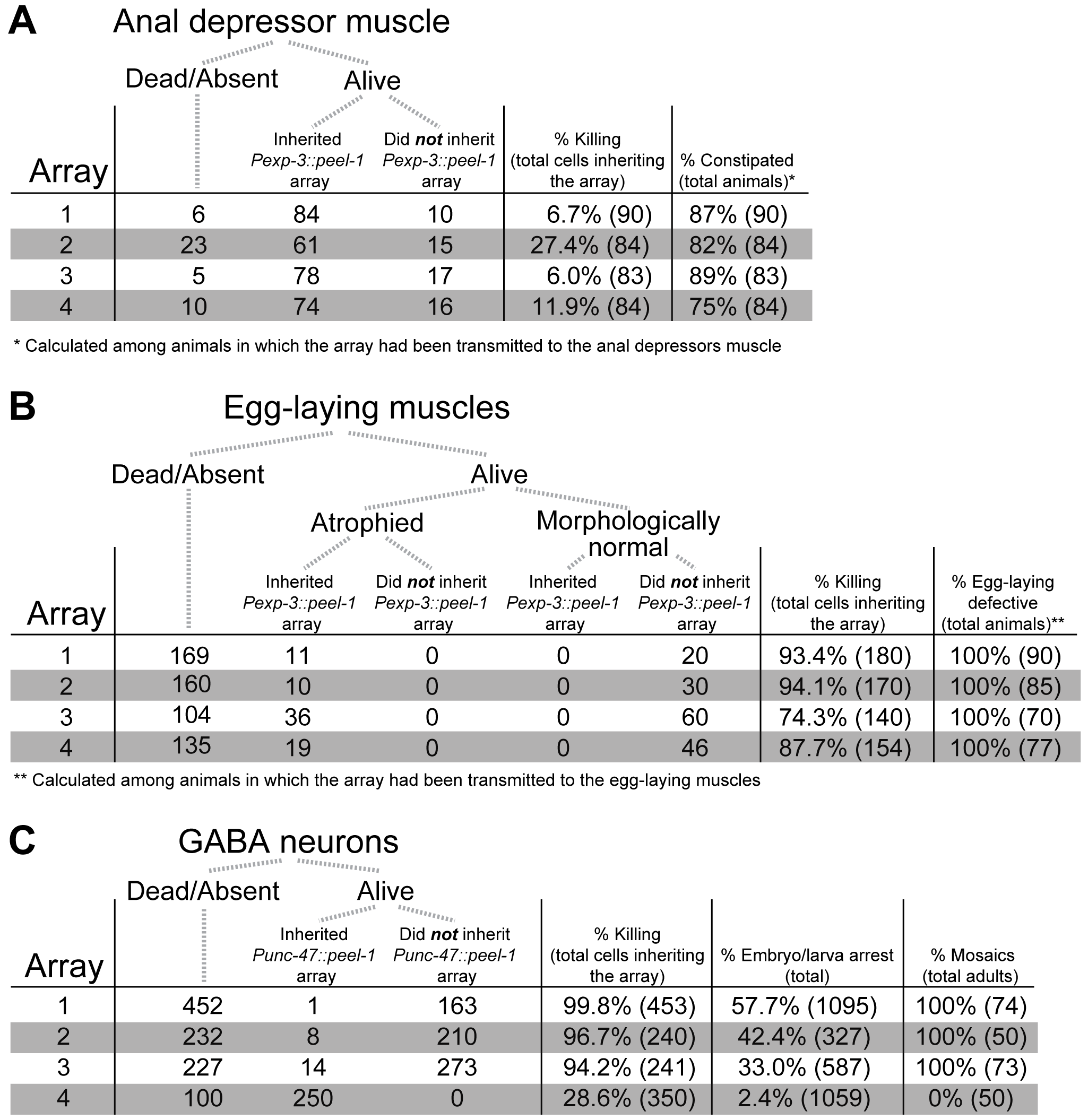

Supplement: Figure S9 — Cell-specific killing via ectopic expression of peel-1. (A–B) The Pexp-3::peel-1 arrays were crossed to a strain carrying an insertion of Pmyo-3::GFP, which serves as a marker of the egg-laying muscles and the anal depressor muscle. Live muscle cells were classified as inheriting the array if they expressed the co-injection marker, Pmyo-3::mCherry. One hundred animals were scored for each array, and two of the four egg-laying muscles were scored per animal. (C) The Punc-47::peel-1 arrays were crossed to a strain carrying an insertion of Punc-47::GFP, which serves as a marker for the GABA neurons. Live neurons were classified as inheriting the array if they expressed the co-injection marker, Prab-3::mCherry. For each array, 50–74 animals were scored, and 6 to 10 neurons were scored per animal. (TIF) [file pbio.1001115.s009.tif]
